# Comparative analysis of mitochondrial genomes of *Schisandra repanda* and *Kadsura japonica*

**DOI:** 10.3389/fpls.2023.1183406

**Published:** 2023-07-03

**Authors:** Hyo Ju Lee, Yi Lee, Sang-Choon Lee, Chang-Kug Kim, Ji-Nam Kang, Soo-Jin Kwon, Sang-Ho Kang

**Affiliations:** ^1^ Genomics Division, National Institute of Agricultural Sciences, Jeonju, Republic of Korea; ^2^ Department of Industrial Plant Science and Technology, Chungbuk National University, Cheongju, Republic of Korea; ^3^ Phyzen Genomics Institute, Seongnam, Republic of Korea

**Keywords:** *Schisandra repanda*, *Kadsura japonica*, mitogenome, genome assembly, InDel markers

## Abstract

The family Schisandraceae is a basal angiosperm plant group distributed in East and Southeast Asia and includes many medicinal plant species such as *Schisandra chinensis*. In this study, mitochondrial genomes (mitogenomes) of two species, *Schisandra repanda* and *Kadsura japonica*, in the family were characterized through *de novo* assembly using sequencing data obtained with Oxford Nanopore and Illumina sequencing technologies. The mitogenomes of *S. repanda* were assembled into one circular contig (571,107 bp) and four linear contigs (10,898–607,430 bp), with a total of 60 genes: 38 protein-coding genes (PCGs), 19 tRNA genes, and 3 rRNA genes. The mitogenomes of *K. japonica* were assembled into five circular contigs (211,474–973,503 bp) and three linear contigs (8,010–72,712 bp), with a total of 66 genes: 44 PCGs, 19 tRNA genes, and 3 rRNA genes. The mitogenomes of the two species had complex structural features with high repeat numbers and chloroplast-derived sequences, as observed in other plant mitogenomes. Phylogenetic analysis based on PCGs revealed the taxonomical relationships of *S. repanda* and *K. japonica* with other species from Schisandraceae. Finally, molecular markers were developed to distinguish between *S. repanda*, *K. japonica*, and *S. chinensis* on the basis of InDel polymorphisms present in the mitogenomes. The mitogenomes of *S. repanda* and *K. japonica* will be valuable resources for molecular and taxonomic studies of plant species that belong to the family Schisandraceae.

## 1 Introduction

Mitochondria are essential organelles that play an important role in energy production through aerobic respiration in most eukaryotic cells (Newton, 1988). They also regulate vital activities *in vivo* by participating in metabolic processes such as cell differentiation, apoptosis, cell growth, and cell division (Kroemer and Reed, 2000; van Loo et al., 2002; Bonora and Pinton, 2014). Mitochondria arose from endosymbiotic *α*-proteobacteria in archaea-derived host cells and eventually came to possess an independent mitochondrial genome (mitogenome) *via* their evolution into eukaryotic organelles (Gray et al., 1999; Lang et al., 1999).

The mitogenomes of plants are larger and more complicated than those of other eukaryotes. They contain varied master circular, linear, and branching structures (Backert et al., 1997; Rice et al., 2013; Gualberto et al., 2014). Since mitogenomes of *Marchantia polymorpha* and *Arabidopsis thaliana* were first mapped, a total of hundreds land plants mitogenomes have been deposited in the Organelle Genome Resources of NCBI. The mitogenome sizes in land plants vary greatly, from 66 kb (*Viscum scurruloideum*; Skippington et al., 2015) to 11.3 Mb (*Silene conica*; Sloan et al., 2012). Similarly, the number of protein-coding genes (PCGs) ranges from 19 (*Viscum scurruloideum*) to 79 (*Ammopiptanthus mongolicus*; unpublished accession NC_039660). Mitogenome size also differs between related species (Sloan et al., 2012; Gualberto and Newton, 2017). It has diversified because of foreign DNA insertion, nuclear or chloroplast sequence insertion, and recombination activity in non-coding regions such as repeat and intron sequences (Alverson et al., 2011; Gandini and Sanchez-Puerta, 2017; Gualberto and Newton, 2017; Cui et al., 2021).

In land plants such as *Cucurbita pepo* (Alverson et al., 2010), *Cycas taitungensis* (Chaw et al., 2008), *Garcinia mangostana* (Wee et al., 2022), and *Oryza sativa* (Notsu et al., 2002), short repeats of less than 100 bp or large repeats of 1 kb or more contribute significantly to mitogenome size (Arrieta-Montiel et al., 2009; Wynn and Christensen, 2019). At least 300 MYA, when plants first evolved into seed plants, chloroplast DNA containing PCGs and tRNAs frequently transferred into the mitogenome (Wang et al., 2007). Such transfer of non-functional plastid-derived sequences to the mitogenome can introduce new chimeric genes, tRNAs, or promoters (Nakazono et al., 1996; Miyata et al., 1998; Hao and Palmer, 2009; Wang et al., 2012). Furthermore, introns can be acquired by the mitogenome *via* gene transfer (Palmer et al., 2000), and AT contents can be increased even in species with relatively abundant GCs in the genome (Sloan and Wu, 2014). These characteristics provide useful information for the study of plant evolution and phylogeny (Wallace et al., 1988; Cameron et al., 2007; Mower et al., 2012).

The family Schisandraceae is composed of basal angiosperm plants of the order Austrobaileyales and branches out after Amborellales and Nymphaeales (Qiu et al., 1999; Leitch and Hanson, 2002). Schisandraceae plants that belong to the genera *Schisandra* and *Kadsura* are distributed throughout Korea, China, and Japan and are valuable economic and medicinal resources (Liu et al., 2014). The fruits of Schisandraceae have anticancer, antioxidant, and anti-inflammatory properties (Li et al., 2018; Zhou et al., 2021) and contain lignans, polysaccharides, flavonoids, and organic acids (Jiang et al., 2015; Zhou et al., 2021). These features have driven interest in Schisandraceae plants (Liu et al., 2022), and research has been conducted on the extraction of fruit-derived substances (Lee and Kim, 2010; Nam et al., 2014; Yi et al., 2016), individual breeding techniques (Kim et al., 2014; Boo and Kim, 2020), flower growth, and sex determination (Liu et al., 2022). In addition, genes involved in compound biosynthesis have been recently identified using transcriptome and metabolome analyses (Chun-Yu et al., 2020; Hong et al., 2022; Li et al., 2022). However, it is difficult to distinguish between *Schisandra* species because the dried mature fruits are morphologically similar (Lee et al., 2013). Furthermore, Schisandraceae plant classification is complex and has changed several times over the last few decades (Li et al., 2018).

In this study, we aimed to gain a better understanding of mitogenome characteristics, structure, evolution, and function. We therefore assembled the mitogenomes of *Schisandra repanda* and *Kadsura japonica* using Nanopore and Illumina sequencing. We used the resulting assembled mitogenomes to conduct taxonomic studies and investigate the relationships between highly utilized Schisandraceae species. Our results will help protect these species and develop their genetic resources.

## 2 Materials and methods

### 2.1 DNA extraction and sequencing

Fresh leaf samples were collected from single *S. repanda* and *K. japonica* plant growing at Jeju Agricultural Research Field, and genomic DNA was extracted. For Illumina sequencing, 200 ng genomic DNA was sonicated to a fragment size of 350 bp using a Covaris S2 system (Covaris, USA) and processed with the Illumina TruSeq Nano Sample Prep kit (Illumina, USA) according to the manufacturer’s instructions. The constructed library was quantified using digital PCR and Taqman Probe (Thermo Fisher Scientific, USA) and sequenced using an Illumina NovaSeq 6000 platform. For Oxford Nanopore sequencing, 10 µg unfragmented genomic DNA was processed using the Quick Ligation Kit (NEB, USA) and SQK-LSK109 Ligation kit (ONT, UK) according to the manufacturers’ instructions. The purified library was loaded into a MinION Flow Cell (ONT, UK) and sequenced for 72 h.

### 2.2 Mitogenome assembly and annotation

The quality trim tool (Phred score > 20) in the CLC Assembly Cell package (ver. 4.2.1, Qiagen, Denmark) and Porechop software (ver. 0.2.3; https://github.com/rrwick/Porechop) with default parameters were used to trim the Illumina and Nanopore raw sequencing data and remove adaptor and chimeric sequences.

The trimmed Nanopore data were assembled using the NextDenovo program (ver. 2.3.1; https://github.com/Nextomics/NextDenovo) with 1-kb read cutoff and 100-Mb genome size as the parameters. The total assembled contigs were aligned with mitochondrial genome sequence of *Schisandra chinensis* (MK860624) using nucmer (ver. 4.0.0beta2; https://github.com/mummer4/mummer) with default parameters and then contigs that were aligned with the mitochondrial genome sequence were selected as mitochondrial contigs. The selected contigs were merged, gap-filled, and error-corrected by a series of mapping the trimmed Illumina data using clc_ref_assemble and clc_mapping_viewer with default parameters in CLC Assembly Cell package ver. 4.2.1 (QIAGEN, Denmark) (Kim et al., 2015).

The assembled mitogenome sequences were validated by read mapping of Nanopore and Illumina sequencing data. The trimmed reads were mapped to the assembled mitogenome sequences, and the consistency and connectivity of the mapped reads on the mitogenome sequences and junctions between contigs were confirmed using clc_ref_assemble, clc_mapping_viewer, and clc_mapping_info with default parameters in CLC Assembly Cell package ver. 4.2.1 (QIAGEN, Denmark).

The mitogenome sequences were annotated using Artemis (Carver et al., 2012) and GeSeq (Tillich et al., 2017) programs on the basis of similarity with the mitochondrial reference genome (*S. chinensis* chromosome 1 mitochondrion, complete sequence [MK860624]) (Baek et al., 2019). In addition, manual curation with BLAST was used to pinpoint particular gene areas.

Circular maps of the mitogenome were created with the annotated data using OGDRAW (http://ogdraw.mpimp-golm.mpg.de; Lohse et al., 2007).

### 2.3 Repeat sequence analysis

Repeat sequences, such as forward and reverse repeats, in mitogenomes were identified using the REPuter program (ver. 2.3.0), with maximum size of gaps between repeat instances of 30 (Kurtz et al., 2001). Simple sequence repeats (SSRs) were detected using the MISA program (ver. 2.1; https://webblast.ipk-gatersleben.de/misa/), with mononucleotide, dinucleotide, trinucleotide, tetranucleotide, pentanucleotide, and hexanucleotide repeat parameters set as 10, 5, 3, 4, 3, and 3, respectively (Beier et al., 2017). Tandem repeats were found using the Tandem Repeats Finder program (ver. 4.09; https://tandem.bu.edu/trf/trf.html), with min match = 2, min mismatch = 7, min score = 50, and max period = 2,000 (Benson, 1999).

### 2.4 Identification plastid-derived sequences in mitochondrial DNA

The mitogenome sequences of *S. repanda* and *K. japonica* were examined for the presence of plastid-derived sequences using BLASTn with megaBlast parameters and a cutoff e-value of 1e^–5^ against the *S. chinensis* chloroplast genome sequence (NC_034908).

### 2.5 RNA-editing analysis

PREPACT (ver. 3.12.0; http://www.prepact.de/prepact-main.php) with default options was utilized to predict RNA editing sites within PCGs of *S. repanda* and *K. japonica* mitogenomes. The cutoff value was set to 0.001 to ensure prediction accuracy (Lenz et al., 2010; Lenz and Knoop, 2013; Lenz et al., 2018).

### 2.6 Ka/Ks analysis

The multiple sequence alignments of 33 commonly found genes from four species were performed using MAFFT v7.305b and the alignments were corrected with Gblocks v0.91b94, and then we used ParaAT v2.0 was used to estimate the Ka/Ks ratio (Castresana, 2000; Zhang et al., 2012; Katoh and Standley, 2013). The plot was drawn using in-house Python and R scripts.

### 2.7 Phylogenetic analysis

Phylogenetic analysis was performed using 14 conserved coding sequences (CDSs; *atp1*, *atp6*, *atp8*, *atp9*, *cob*, *cox2*, *mttB*, *nad1*, *nad2*, *nad3*, *nad5*, *nad6*, *rpl5*, and *rps12*) in 17 species. Concatenated CDSs were aligned using the MAFFT program (ver. 7) with default parameters and used as input data for the phylogenetic analysis (Katoh et al., 2019). The RAxML program (ver. 8.2.12) was used for the phylogenetic analysis, with the substitution model GTR + I + G, and 1,000 bootstrap replicates (Stamatakis, 2014). The FigTree program (ver. 1.4.3; http://tree.bio.ed.ac.uk/software/figtree/) was used to visualize the trees.

### 2.8 Gene clusters conserved in the mitogenomes

Gene orders in the mitogenomes of *S. repanda* and *K. japonica* were compared with those in the mitogenomes of 15 other species. Two nearby genes with no intervening genes and less than 5 kb of intergenic sequences between them were defined as clusters. A gene cluster was considered conserved when the two flanking genes in the *S. repanda* and *K. japonica* mitogenomes were also present in other mitogenomes.

### 2.9 Identification of InDel polymorphisms and PCR amplification

The mitogenomes of *S. repanda*, *K. japonica*, and *S. chinensis* were compared reciprocally using megaBLAST with a cutoff e-value of 1e^–5^ and minimum length of 10 kb. Based on the megaBLAST results, homologous regions of >10 kb among the three mitogenomes were selected and InDel polymorphisms were identified. InDels of >5 bp were selected and used to design InDel primers. The flanking sequences ( ± 300 bp) of the selected InDels were extracted from *S. chinensis* mitogenome sequences and used to design primer sets for PCR amplification of the InDel sites. The PCR primers were designed using Primer 3 software with modified parameters: primer size of 17–25 mer, temperature of 50–60°C, GC of 45–55, and amplicon size of 300–500 bp (Untergasser et al., 2012). The specificity of the designed primers was confirmed using BLASTN against *S. chinensis* mitogenome sequences. PCR conditions were 5 min at 95°C for pre-denaturation; 40 cycles of 30 s each at 95°C, 58°C, and 72°C; and a final extension for 5 min at 72°C. Gel electrophoresis on 4% agarose gels in TAE buffer with loading dye was used to analyze the PCR products.

## 3 Results

### 3.1 Whole-genome sequencing and assembly of the *S. repanda* and *K. japonica* mitogenomes

The mitogenomes of *S. repanda* and *K. japonica* were assembled using sequencing data obtained from the third- and next-generation sequencing platforms of Oxford Nanopore and Illumina technology, respectively. Additionally, we confirmed the assembly sequences through read mapping and checking read depth (**Supplementary figures 1, 2**). For *S. repanda*, 10.79 and 2.69 Gb were obtained by trimming 10.82 Gb of raw Nanopore data and 3.02 Gb of raw Illumina data, respectively. For *K. japonica*, 9.80 and 1.99 Gb were obtained by trimming 9.84 Gb of raw Nanopore data and 2.27 Gb of raw Illumina data, respectively (**Supplementary table 1**). The trimmed sequencing data were then *de novo* assembled to characterize the mitogenomes of the two species.

Five and eight mitochondrial contigs with varying genome sizes and gene contents were assembled from *S. repanda* and *K. japonica*, respectively. The *S. repanda* mitogenome had one circular and four linear contigs: (circular-1: 571,107 bp; linear-1: 607,403 bp; linear-2: 215,128 bp; linear-3: 42,796 bp; and linear-4: 10,898 bp; **Supplementary figure 3**). We investigated gene content (PCGs, tRNA, and rRNA) in these five *S. repanda* mitochondrial contigs: *S. repanda* circular-1 included 34 genes (18 PCGs, 13 tRNAs; 3 rRNAs), linear-1 contained 34 genes (20 PCGs, 11 tRNAs, 3 rRNAs), linear-2 contained 11 genes (4 PCGs, 4 tRNAs, 3 rRNA), linear-3 contained 4 PCGs, and linear-5 contained 2 PCGs (**Supplementary table 2**). In all, 60 genes were identified, comprising 38 protein-coding (23 unique core genes except *nad9* and 15 variable genes), 19 tRNA, and 3 rRNA genes (**Table 1**).

**Table 1:** Annotated genes in the mitogenomes of *S. repanda* and *K. japonica*.

Group of genes	Common genes	Unique genes			
		*S. repanda*		*K. japonica*	
Complex I (NADH dehydrogenase)	*nad1*, *nad2*, *nad3*, *nad4*, *nad4L*, *nad5*, *nad6*, *nad7*			*nad9*	
Complex II (Succinate dehydrogenase)	*sdh3*	–		*sdh4*	
Complex III (Ubiquinol-cytochrome c reductase)	*cob*	–		–	
Complex IV (Cytochrome c oxidase)	*cox1*, *cox2*, *cox3*	–		–	
Complex V (ATP synthase)	*atp1*, *atp4*, *atp6*, *atp8*, *atp9*	–		–	
Cytochrome c biogenesis	*ccmB*, *ccmC*, *ccmFc*, *ccmFn*	–		–	
Large subunit ribosomal proteins	*rpl2*, *rpl5*, *rpl10*, *rpl16*	–		–	
Small subunit ribosomal proteins	*rps2*, *rps3*, *rps7*, *rps10*, *rps11*, *rps12*, *rps13*, *rps14*, *rps19*	–		*rps1*, *rps4*	
Maturase	*matR*	–		–	
Transport membrane protein	*mttB*	–		–	
Ribosomal RNAs	*rrn5*, *rrnL*, *rrnS*	–		–	
Transfer RNAs	*trnA-UGC*, *trnC-GCA*, *trnD-GUC*, *trnE-UUC*, *trnF-GAA*, *trnG-GCC*, *trnH-GUG*, *trnK-UUU*, *trnL-CAA*, *trnM-CAU*, *trnN-GUU*, *trnP-UGG*, *trnR-UCU*, *trnS-GCU*, *trnW-CCA*, *trnY-GUA*	*trnG-UCC*, *trnI-GAU*, *trnU-GGU*		*trnL-UAG*, *trnQ-UUG*, *trnV-GAC*	
Other genes	*petA*	–		*accD*, *rbcL*, *ycf4*	

The mitogenome of *K. japonica* had five circular and three linear contigs: (circular-1: 973,503 bp; circular-2: 897,204 bp; circular-3: 848,837 bp; circular-4: 261,590 bp; circular-5: 211,474 bp; linear-1: 72,712 bp; linear-2: 68,176 bp; and linear-3: 8,010 bp; **Supplementary figure 4**). *K. japonica* circular-1 included 45 genes (29 PCGs, 13 tRNAs, 3 rRNAs), circular-2 included 43 genes (28 PCGs, 12 tRNAs, 3 rRNAs), circular-3 included 43 genes (27 PCGs, 13 tRNAs, 3 rRNAs), circular-4 included 14 genes (7 PCGs, 7 tRNAs), circular-5 included 9 genes (7 PCGs, 2 tRNA), linear-1 contains 3 genes (1 PCG, 2 tRNAs), linear-2 contained 6 genes (5 PCGs, 1 tRNA), and linear-3 contained only 1 PCG (**Supplementary table 2**). A total of *K. japonica* 66 genes were identified, including 44 protein-coding (24 unique core genes and 20 variable genes), 19 tRNA, and 3 rRNA genes (**Table 1**).

**Figure 1:**
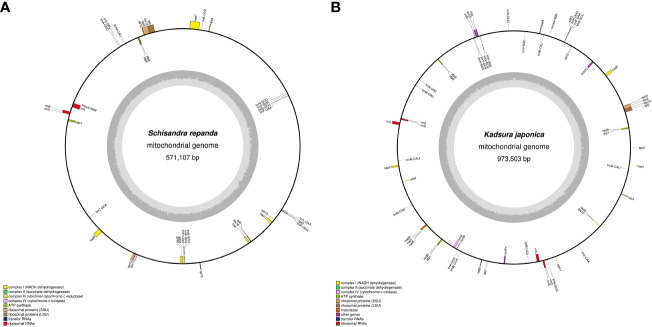
Maps of *S. repanda* and *K. japonica* circular-1 mitogenomes. **(A)** The *S. repanda* mitogenome has a total length of 571,107 bp. **(B)** The *K. japonica* mitogenome has a total length of 973,503 bp. Mitochondrial gene functional groups are represented by the same color.

Based on the assembled contig sequences, we performed a BLASTN search to look for regions of similarity between contigs. As a result, 18 (1,054-43,663 bp) and 72 (1,041-819,626 bp) similar sequences were identified in *S. repanda* and *K. japonica*, respectively (**Supplementary table 3**; **Supplementary figure 5 and 6**). In particular, the *K. japonica* circular -1 to -3 sequences 815,430 bp, 815,483 bp, and 819,626 bp were identified as the longest similar sequences. Many of the ends of the linear contigs are homologous to other genomic regions (**Supplementary figure 5 and 6**), suggesting a multipartite structure of these genomes. The mitogenomes of five contigs of *S. repanda* and eight contigs of *K. japonica* have been deposited in NCBI under GenBank Accession Numbers OK077167–OK077171 and OK077159–OK077166, respectively. *S. repanda* circular-1 (Accession No. OK077168) and *K. japonica* circular-1 (Accession No. OK077159) were used in all further analyses (repeat sequence analysis and DNA migration from chloroplasts to mitochondria), except prediction of RNA-editing, Ka/Ks, phylogenetic analysis, gene clusters, and identification of InDel, as they are the largest circular and contain the most genes (**Figure 1**).

### 3.2 Repeat sequence analysis

**Figure 2:**
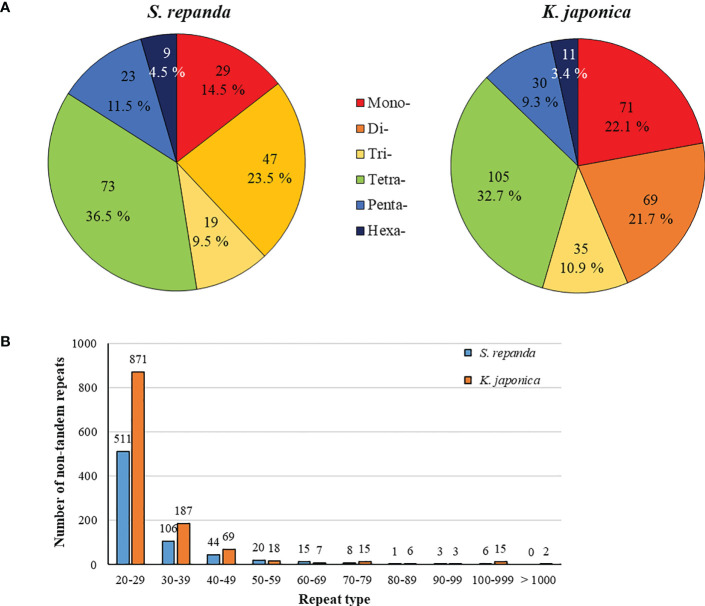
Frequency distribution of SSRs and non-tandem repeats in *S. repanda* and *K. japonica* mitogenomes. **(A)** Number of SSRs. Different types of SSRs are represented by different colors. **(B)** Number of non-tandem repeats.

SSRs are DNA sequences in eukaryotic genomes that typically consist of 1- to 6-bp nucleotides (Lovin et al., 2009). SSR frequency varies among plant species, although SSRs are uniformly dispersed throughout the mitogenome (Zhang et al., 2020). MISA program analysis revealed that the SSR distribution ratios of *S. repanda* and *K. japonica* were highly comparable (**Figure 2A**; **Supplementary table 4**). *S. repanda* and *K. japonica* had 200 and 320 SSRs, respectively; the proportion of tetranucleotide repeats was the highest at 36.5% and 32.7%, and the proportion of hexanucleotide repeats was the lowest at 4.5% and 3.4%, respectively.

Analysis of pentanucleotide and hexanucleotide locations revealed four SSRs in *S. repanda* located in *nad5*, *nad7*, and *rpl5* and two SSRs in *K. japonica* located in *rpl2* and *rps3* (**Supplementary table 5**). The Tandem Repeats Finder program was used to analyze perfect tandem repeats, with 11 and 24 identified in *S. repanda* and *K. japonica*, respectively (**Supplementary table 6**). Among them, *rpl2*, *rrnL*, and *nad5* in *S. repanda* had three tandem repeats, and *rrnL* in *K. japonica* had two tandem repeats. The SSRs and tandem repeat sequences were mostly in the intergenic spacers.

Using the REPuter program to analyze non-tandem repeats other than SSRs and tandem repeats, 714 (total length, 21,695 bp; 3.8%) and 1,193 (total length, 53,852 bp; 5.53%) repeats were detected in *S. repanda* and *K. japonica*, respectively (**Supplementary table 7**). The most abundant repeats in *S. repanda* and *K. japonica* were 511 and 871 repeats in the 20- to 29-bp range, with the longest repeats measuring 539 and 12,605 bp, respectively (**Figure 2B**). Non-tandem repeats were similarly distributed in the two species, and only *K. japonica* had two long repeat sequences of more than 1 kb. According to a repeat analysis, *K. japonica* had longer repeats than *S. repanda*.

### 3.3 DNA migration from chloroplasts to mitochondria

**Figure 3:**
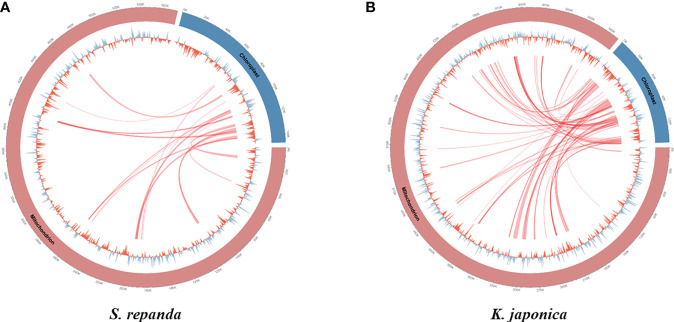
Representation of gene transfers between mitochondrial and chloroplast genomes for *S. repanda* and *K. japonica*. Regions of chloroplast sequences that have been inserted into the mitogenome are indicated by red lines. **(A)** *S. repanda*. **(B)** *K. japonica*.

Intergenomic gene transfer is the movement of DNA sequences between mitochondria, chloroplasts, and the nucleus, as opposed to DNA sequence movement by inheritance, and it is an important cause of mitogenome expansion and evolution (Adams et al., 2001; Renner and Bellot, 2012; Hao et al., 2022). We compared the complete chloroplast sequence of *S. chinensis* (NC_034908.1) to the *S. repanda* and *K. japonica* mitogenomes and identified 16 (32–2,807 bp, 3.39%) and 37 (39–7,515 bp, 4.34%) fragments with high similarities to chloroplast genome, respectively (**Figure 3**; **Supplementary table 8**). There were eight complete tRNAs (*trnH-GTG*, *trnL-CAA*, *trnM-ATG*, *trnN-GUU*, *trnP-TGG*, *trnR-TCT*, *trnV-GAC*, and *trnW-CCA*) and three partial tRNAs (*trnH-GTG*, *trnN-GUU*, and *trnR-TCT*). The chloroplast-derived sequences also included two partial rRNAs (*rrn16s* and *rrn23S*) and 29 partial PCGs (*accD*, *atp1*, *atpA*, *atpE*, *atpF*, *atpH*, *cemA*, *infA*, *ndhB*, *ndhI*, *petA*, *petD*, *psaA*, *psaI*, *psaJ*, *psbE*, *rbcL*, *rpl20*, *rpl33*, *rpl36*, *rpoA*, *rpoC1*, *rpoC2*, *rps2*, *rps7*, *rps8*, *ycf1*, *ycf2*, and *ycf4*).

### 3.4 Prediction of RNA editing sites

RNA editing, i.e., the post-transcriptional addition, deletion, or conversion of nucleotides in the coding region of transcribed RNA, occurs frequently in the plant mitogenome (Gray, 2003; Yang et al., 2017). In the PCGs of *S. repanda* and *K. japonica* mitogenomes, we detected 639 and 684 RNA editing sites, respectively (**Supplementary table 9**). The first base position of the codon had 224 and 237 RNA editing sites, and the second base position had 415 and 447, respectively, in *S. repanda* and *K. japonica.* No RNA-editing sites were predicted at the third base position (**Supplementary table 10**). To date, the RNA-editing prediction programs cannot reliably predict RNA-editing at third base positions due to technical limitation. After RNA editing, 28.79% and 29.39% of the amino acids remained hydrophobic, whereas 13.3% remained hydrophilic in *S. repanda* and *K. japonica*. However, 47.42% and 47.37% of the amino acids were converted from hydrophilic to hydrophobic, and 9.86% and 9.36% were converted from hydrophobic to hydrophilic in *S. repanda* and *K. japonica*, respectively; only four of them were changed to stop codons in the *S. repanda* and *K. japonica* mitogenome. After RNA editing, leucine was the predominant amino acid in 48.67% of *S. repanda* and 41.96% of *K. japonica*, respectively.

### 3.5 The substitution rates of protein-coding genes

**Figure 4:**
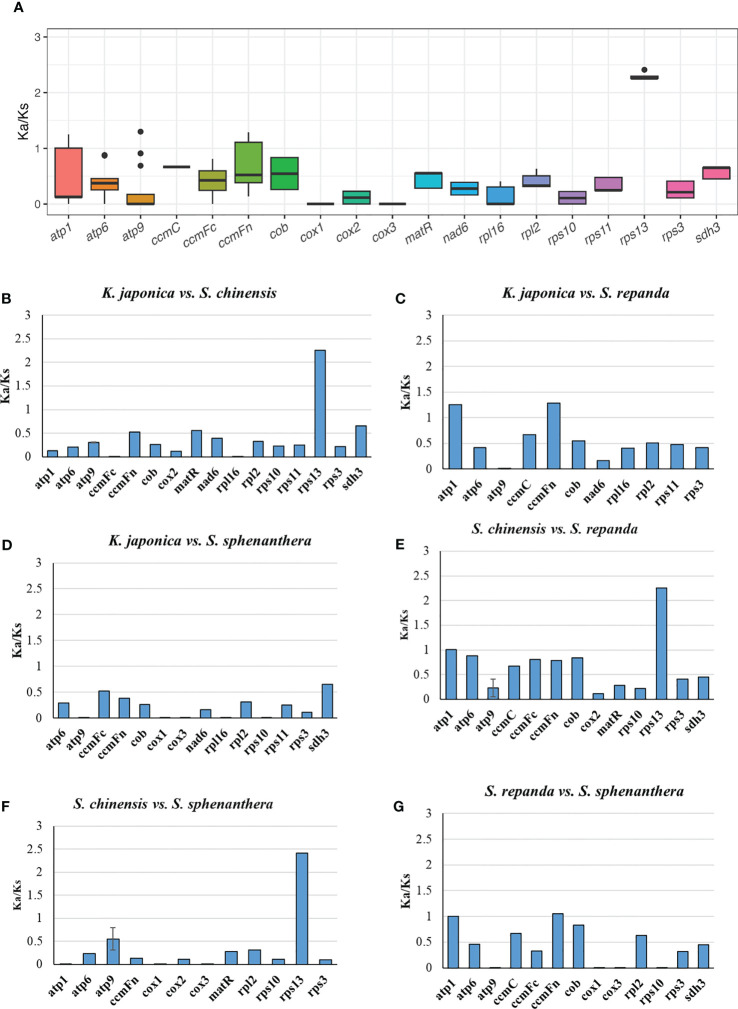
The Ka/Ks values of 33 PCGs of *S. repanda*, *K. japonica*, *S. chinensis*, and *S. sphenanthera*. **(A)** Box plot for pairwise divergence of Ka/Ks ratio (mean ± SD, and range). **(B-G)** Bar graph for pairwise divergence of Ka/Ks ratio (error bars representing either the mean ± SD). The missing values, such as *atp1* in *S. repanda vs S. sphenanthera*, could be due to the limitation of the ParaAT, Ka/Ks cannot be calculated when sequences are identical between species.

The number of non-synonymous substitutions (Ka) and synonymous substitutions (Ks) is important for phylogenetic reconstruction of related species and for understanding the evolutionary dynamics of protein coding sequences (Fay and Wu, 2003). Ka/Ks values are used to determine whether certain protein-coding genes have undergone selection pressure during evolution. Ka/Ks=1 indicates neutral evolution, Ka/Ks > 1 indicates positive selection, and Ka/Ks <1 indicates negative selection (Zhang et al., 2006). In this study, all of the 33 PCGs of *S. repanda*, *K. japonica*, *S. chinensis*, and *S. sphenanthera* mitogenome were used to calculate the Ka and Ks substitution rates. As a result, due to the limitation of the ParaAT, Ka/Ks cannot be calculated when sequences are identical between species. In the comparison of the four species, the Ka/Ks of *rps13* was the highest at 2.26 to 2.42, and appeared to be under positive selection along with genes such as *atp9* (**Figure 4A**; **Supplementary table 11**). Genes such as *ccmFc*, *cob*, *cox2*, *matR*, *nad6*, *sdh3*, *RPL* and *RPS* showed Ka/Ks values less than 1, indicating that they were under negative selection (**Figure 4B-G**). The *cox1* and *cox3* exhibited the lowest Ka/Ks ratio, suggesting that these genes had fewer changes and well conserved between species.

### 3.6 Phylogenetic analysis

**Figure 5:**
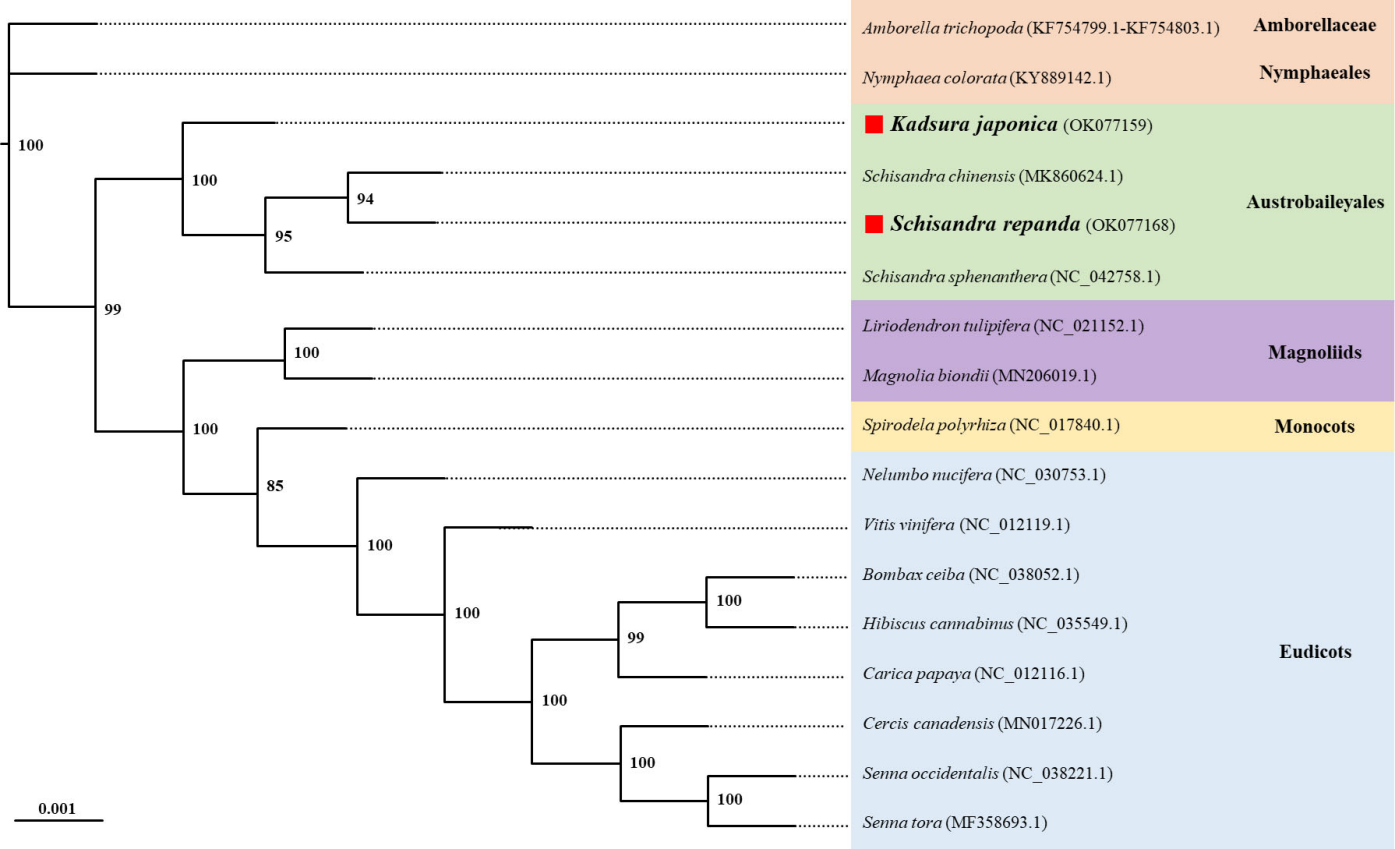
Phylogenetic relationships of *S. repanda* and *K. japonica* with other 15 plant species. The numbers next to the branches indicate bootstrap values of maximum likelihood phylogenetic trees. GenBank accession numbers are listed after the scientific names. Positions of *S. repanda* and *K. japonica* are indicated in bold.

Phylogenetic analysis using the maximum likelihood (ML) method was performed using a dataset of 14 PCGs from the following 17 plant taxa (including four species from Schisandraceae): *Amborella trichopoda*, *Nymphaea colorata*, *S. chinensis*, *S. repanda*, *K. japonica*, *Schisandra sphenanthera*, *Liriodendron tulipifera*, *Magnolia biondii*, *Spirodela polyrhiza*, *Nelumbo nucifera*, *Vitis vinifera*, *Bombax ceiba*, *Hibiscus cannabinus*, *Carica papaya*, *Cercis canadensis*, *Senna occidentalis*, and *Senna tora* (**Figure 5**). The ML tree contained 15 nodes, of which 14 had at least 90% of the support value with 100% for 10 and 85% for only one; *S. repanda* and *K. japonica* were classified into one clade with bootstrap support values of 100 and 94. The four plants of the family Schisandraceae were placed quite close to each other, with *S. repanda* being the closest to *S. chinensis*. Finally, Schisandraceae plants were classified as basal angiosperms along with *Amborella* and the Nymphaeales, and the placement indicated that the family evolved from mesangiosperms into monocots and eudicots.

### 3.7 Conservation of gene clusters

**Figure 6:**
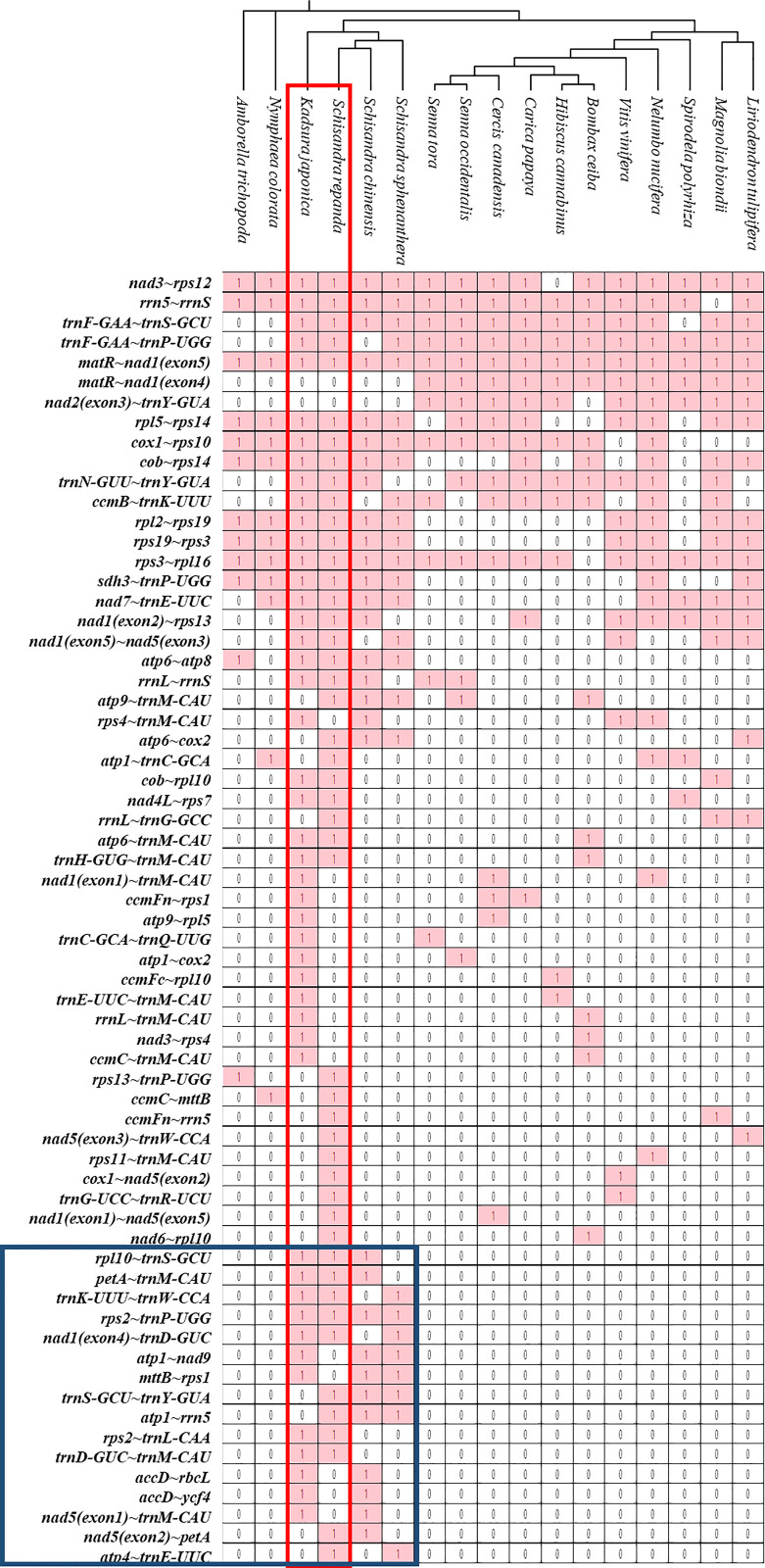
Gene clusters in the mitogenomes of 17 species. Gene sequences of *S. repanda* and *K. japonica* mitogenomes were compared with those of the other 15 species. The evolutionary relationships of the mitogenomes are indicated in the phylogenetic tree. ‘1’ indicates the presence of the gene in the mitogenome. ‘0’ indicateds the absence of the gene in the mitogenomes.

The sequences of rRNA and PCGs in plant mitogenomes are highly conserved, but the relative order of genes is frequently rearranged by homologous recombination. However, some gene clusters have been conserved during evolution (Palmer et al., 2000; Richardson et al., 2013; Gui et al., 2016). There were 47 and 46 conserved gene clusters in all *S. repanda* and *K. japonica* contigs, respectively (**Figure 6**). Three gene clusters (*rpl16~rps3*, *rps3~rps19*, and *rps19~rpl2*) can be traced back to mitochondrial origins in endosymbiotic bacterial ancestors (Takemura et al., 1992; Niu et al., 2022), two gene clusters (*nad3~rps12* and *cox1~rps10*) could be found in gymnosperms, and the *sdh3~trnP-UGG* cluster arose during bryophyte evolution (Turmel et al., 2007). In addition, *trnF-GAA~trnS-GCU* and *trnF-GAA~trnP-UGG* arose during evolution into Schisandraceae and Mesangiospermae. However, sixteen gene clusters (blue box; *rpl10~trnS-GCU*, *petA~trnM-CAU*, *trnK-UUU~trnW-CCA*, *rps2~trnP-UGG*, *nad1*(exon4)~*trnD-GUC*, *atp1~nad9*, *mttB~rps1*, *trnS-GCU~trnY-GUA*, *atp1~rrn5*, *rps2~trnL-CAA*, *trnD-GUC~trnM-CAU*, *accD~rbcL*, *accD~ycf4*, *nad5*(exon1)*~trnM-CAU*, *nad5*(exon2)*~petA*, *atp4~trnE-UUC*) were regained in only Schisandraceae and were confirmed to be lost in the subsequent evolution process. The gene clusters of *nad2*(exon3)*~trnY-GUA* was not present in basal angiosperms, but they appeared to have been regained in the course of evolution in Mesangiospermae, except for *Bombax ceiba*.

### 3.8 Species identification with InDel markers

**Figure 7:**
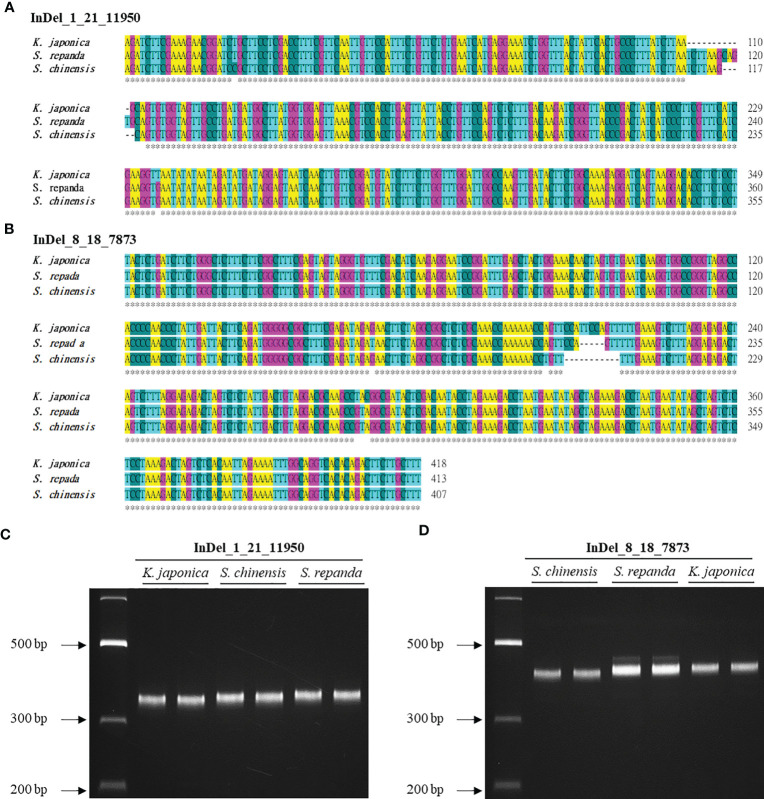
Development of InDel markers for *S. repanda*, *K. japonica*, and *S. chinensis* mitogenomes. **(A, B)** Alignment of the InDel regions. **(C)** PCR amplicons for *K. japonica*, *S. chinensis*, and *S. repanda* using InDel_1_21_11950 were 349, 355, and 360 bp, respectively. **(D)** The InDel_8_18_7873 primer set was used to obtain PCR results for *S. chinensis*, *S. repanda*, and *K. japonica* (407, 413, and 418 bp, respectively).

InDel regions are widely utilized to create markers to distinguish between species because they can be easily detected (Sebbenn et al., 2019). However, *Schisandra* and *Kadsura* species have not been identified using this method on the basis of their mitogenomes. We used BLAST to search for InDel regions of the *S. chinensis* mitogenome in the *S. repanda* and *K. japonica* mitogenomes. InDel markers were designed for 95 of the 130 InDel regions confirmed to be longer than 5 bp (**Supplementary table 12**). We selected two InDel markers for PCR analysis (**Figure 7A, B**; **Supplementary table 13**). Notably, *S. repanda*, *K. japonica*, and *S. chinensis* could be distinguished using PCR amplification with the two primer sets. Based on the *S. chinensis* sequence, 6 and 11 bp were inserted into *S. repanda* and *K. japonica*, respectively, in the case of InDel_8_18_7873, and 5 bp were inserted to *S. repanda* and 6 bp were deleted from *K. japonica* in the case of InDel_1_21_11950 (**Figure 7C, D**). The three species can be distinguished readily using two InDel markers developed through this study.

## 4 Discussion

The mitogenome comprises repeated sequences and recombination regions, leading to a range of sizes and structures, whereas the plant chloroplast genome is made up of a single-ring structure. This difference in complexity has likely contributed to a lack of research into mitogenomes (Mower et al., 2012; Gualberto et al., 2014). Recently, mitogenome research has advanced because of the development of efficient, inexpensive, and precise genome sequencing tools (Han et al., 2022; Wee et al., 2022; Yang et al., 2022). In this study, *S. repanda* and *K. japonica*, two important medicinal plants, were examined for their mitogenome features. According to our sequencing data, *S. repanda* and *K. japonica* have five and eight mitogenome structures, respectively, which is not surprising given the various sizes and structures of the plant mitogenome. For example, five circular mitogenomes have been found in *A. trichopoda* (Rice et al., 2013), and fluorescence microscopy examination of lettuce revealed linear, branching, and circular mitogenome structures (Kozik et al., 2019).

Mitogenomes in angiosperms encode 24 core protein-coding genes, the majority of which are respiratory protein genes: *atp1*, *atp4*, *atp6*, *atp8*, *atp9*, *ccmB*, *ccmC*, *ccmFc*, *ccmFn*, *cob*, *cox1*, *cox2*, *cox3*, *matR*, *mttB*, *nad1*, *nad2*, *nad3*, *nad4*, *nad4L*, *nad5*, *nad6*, *nad7*, and *nad9* (Adams et al., 2002). Although the full-length *nad9* gene was not identified in the *S. repanda* mitogenome, some sequences similar to *nad9* were observed in *S. repanda* circular-1, linear-1, and linear-2. Notably, we observed that the *nad9* gene is similarly lacking from the reported mitogenomes (in GenBank) of plants including *Pisum sativum* subsp. *elatius* (MW160422), *Camellia nitidissima* (NC_067639.1 and ON645224), *Kandelia obovata* (NC_069222.1 and OP756530), and *Ipomoea batatas* (NC_068714.1 and OM808941). Thus, we conclude that *nad9* might truly be absent from the *S. repanda* mitogenome. Future work will examine the *S. repanda* nuclear genome for evidence of a potential loss or shift of the *nad9* gene.

Various repeat sequences, including short (<1 kb), long (>1 kb), and tandem repeats, have been found in the mitogenome sequence (Gualberto et al., 2014; Guo et al., 2017). These repeats not only expand the mitogenome but also cause genomic recombination (Dong et al., 2018; Wang et al., 2019). In this study, we identified 200 (2704 bp, 0.47%) and 321 (4,076 bp, 0.42%) SSRs in *S. repanda* and *K. japonica*, respectively, which can be used to develop important markers for species distinction, genetic diversity, and evolution studies (Powell et al., 1996; Morgante et al., 2002; Guang-Xin et al., 2019). The total lengths of perfect tandem repeats in *S. repanda* and *K. japonica* were 269 and 520 bp, respectively, and the longest non-tandem repeats were 539 and 12,605 bp, respectively. These lengths were greater in *K. japonica* (5.53%) than in *S. repanda* (3.8%). The repeat sequences may have increased the *K. japonica* mitogenome size relative to the *S. repanda* mitogenome size.

In the mitochondrial, chloroplast, and nuclear genomes, gene transfer is possible in both directions. (Martin et al., 1998; Cui et al., 2021). Nuclear and plastid DNA sequences can be transferred to the mitogenome, resulting in mitogenome size changes (Sloan et al., 2012). When a foreign gene is inserted into the mitogenome at this time, it is preferentially inserted into the intergenic region (Zhao et al., 2018). The plastid genome rarely integrates foreign genetic material (Zhao et al., 2018), whereas the mitogenome frequently integrates DNA sequences from the nucleus, chloroplast, and other mitogenomes (Rice et al., 2013; Zhao et al., 2018). The length of the DNA integrated into the mitogenome varies on the basis of plant species but is generally within the range of 1–12% of angiosperm plastome sequences (Mower et al., 2012), such as in *G. mangostana* (1.7%) (Wee et al., 2022) and *C. pepo* (11.6%) (Alverson et al., 2010). Similarly, 3.39% and 4.34% integrated DNA fragments were found in *S. repanda* and *K. japonica*, respectively, driving genome size and genetic and evolutionary diversity.

RNA editing is a post-transcriptional process required for plant development and stress response (Zhu et al., 2014; Shi et al., 2016; Tang and Luo, 2018). RNA editing, which occurs most frequently in mitochondria, causes reorganization of the mitochondrial protein structure by forming new start and stop codons *via* C-to-U conversion or altering the RNA structure *via* splicing site changes (Takenaka et al., 2013; Tang and Luo, 2018; Hao et al., 2021). RNA editing sites were first discovered in the wheat cytochrome c oxidase gene (Covello and Gray, 1989); subsequently, more than 400 sites have been found in the genes of many plants, such as *Acer truncatum* (Ma et al., 2022), *Arabis alpina* (Xu and Bi, 2018), *A. thaliana* (Unseld et al., 1997), *O. sativa* (Notsu et al., 2002), *Suaeda glauca* (Cheng et al., 2021), and *Zea mays* (Hoch et al., 1991). In the mitogenome of *K. japonica*, a start codon was formed in *rpl2* and a stop codon was formed in *ccmFC*, *ccmFN*, and *rps11*. In the mitogenome of *S. repanda*, start codons were formed in *atp8*, *rps10*, and *rpl10*, but no stop codon was identified. The detection of RNA editing sites in these mitogenomes provides clues to predict gene functions, as different amino acids are produced (Cheng et al., 2021).

In most plant species, the PCGs of the mitogenome is relatively conserved compared to that of the animal species, so point mutations are very rare and contribute to maintaining function (Gualberto et al., 2014). Ka values for most genes are smaller than Ks values because deleterious mutations are eliminated during natural selection to maintain mitochondrial function (Hurst, 2002). Nevertheless, the *sdh4* gene is only found in *K. japonica*, indicating that *sdh4* is evolutionarily unstable and has frequently been deleted from the mitogenomes (Adams et al., 2001; Petersen et al., 2017).

PCG and rRNA gene sequences are highly conserved, but the relative order of genes often changes because of genome expansion or recombination (Palmer et al., 2000); nevertheless, a number of highly conserved gene clusters can be found (Richardson et al., 2013; Bi et al., 2016; Dong et al., 2018). The *rps3~rpl16* gene cluster is highly conserved and can be traced to the mitochondrial origin of endosymbiotic bacterial ancestors (Takemura et al., 1992; Niu et al., 2022). Most of the gene clusters are shared by species with close evolutionary relationships (Niu et al., 2022). Eight gene clusters were not found in any other plants, except Schisandraceae. This implies significant genome rearrangements led to rapid degeneration of these gene clusters in other plants (Dong et al., 2018). These gene clusters may have been significant in the development of Schisandraceae plants, in contrast to *Amborella* and Nymphaeales.

Mitogenomes are ideal for lineage studies in seed plants because they evolve more slowly than nuclear and chloroplast genomes and undergo recombination less often (Drouin et al., 2008). Recently, genotyping of both plastomes and mitogenomes has enabled us to distinguish between different plant species (Sebbenn et al., 2019; Han et al., 2022; Ma et al., 2022). InDel markers have the advantage of simplifying plant species identification by comparing amplification product sizes from reference genomes with PCR, making them more cost-effective than single-nucleotide polymorphism or SSR markers (Sohn et al., 2017; Chun et al., 2019). To date, only nuclear and chloroplast genome markers have been reported for Schisandraceae plant identification (Kim et al., 2012; Lee et al., 2013; Jeong et al., 2021). Here, we designed markers based on two InDel regions in the mitogenome sequences of *S. repanda* and *K. japonica* compared to *S. chinensis*. These InDel markers could clearly distinguish between the three species of Schisandraceae. In addition, our phylogenetic analysis results will be helpful for future evolutionary research.

## 5 Conclusion

We assembled and analyzed the mitogenomes of *S. repanda* and *K. japonica*. Various forms of repeats and chloroplast-derived sequences in these mitogenome likely caused mitogenome expansion. In addition, we catalogued RNA editing sites, which will help predict novel gene activity that arises from changes in the protein structure. The sixteen gene clusters we identified as specific to Schisandraceae will be useful to study the evolution of this family. Also, *S. repanda*, *K. japonica*, and *S. chinensis* could be distinguished using two InDel markers that we designed. In conclusion, our work on the mitogenomes of *S. repanda* and *K. japonica* offers new avenues for investigating the evolution of Schisandraceae and for understanding the individual species.

## Data availability statement

The datasets presented in this study can be found in online repositories at NCBI, accession numbers: OKO77167-OKO77171.

## Author contributions

S-HK conceived and designed the study. HJL, YL, S-CL, C-KK, J-NK, S-JK prepared the materials and performed related analysis. HJL and S-HK interpreted the data and wrote the manuscript. All authors contributed to the article and approved the submitted version.
