# Supplementary material for: Comparative analysis of mitochondrial genomes of Schisandra repanda and Kadsura japonica
Source: Front Plant Sci. 2023 Jul 3;14:1183406. doi: 10.3389/fpls.2023.1183406 (PMC10352487; doi:10.3389/fpls.2023.1183406)
Supplement: Supplementary file 1 [file DataSheet_1.docx]

Supplementary Material

Comparative analysis of mitochondrial genomes of *Schisandra repanda* and *Kadsura japonica*

Hyo Ju Lee^1^, Yi Lee^2^, Sang-Choon Lee^3^, Chang-Kug Kim^1^, Ji-Nam Kang^1^, Soo-Jin Kwon^1^ and Sang-Ho Kang^1*^

*** Correspondence:** Sang-Ho Kang: hosang93@korea.kr

# Supplementary Tables and Figures

Supplementary Table 1. Statistics of the genome sequencing in *S. repanda* and *K. japonica* mitgenomes.

Supplementary Table 2. Gene profile and organization of the *S. repanda* and *K. japonica* mitogenomes.

Supplementary Table 3. BLASTN results among *S. repanda* and *K. japonica* contig (task option megablast, cutoff e-value 1e-5, match length > 1 kb).

Supplementary Table 4. Statistics on SSRs in the contig of *S. repanda* circular-1 and *K. japonica* circular-1.

Supplementary Table 5. Distribution of penta- and hexa- SSRs in the *S. repanda* circular-1 and *K. japonica* circular-1 contigs.

Supplementary Table 6. Distribution of perfect tandem repeats in the *S. repanda* circular-1 and *K. japonica* circular-1 contigs.

Supplementary Table 7. The distribution of repeats in the *S. repanda* circular-1 and *K. japonica* circular-1 contigs*.*

Supplementary Table 8. Plastid insertions in the mitogenome of *S. repanda* circular-1 and *K. japonica* circular-1 contigs.

Supplementary Table 9. The distribution of RNA editing sites in the *S. repanda* and *K. japonica* PCGs

Supplementary Table 10. Prediction of RNA editing sites in *S. repanda* and *K. japonica* PCGs

Supplementary Table 11. The Ka/Ks values of 33 PCGs of four schisandraceae

Supplementary Table 12. Primer information for the 95 Indel markers developed in this study. Yellow highlights are the primer sets used to distinguish between *S. repanda, K. japonica,* and *S. chienesis.*

Supplementary Table 13. Primer information used in this study to identify *S. repanda, K. japonica,* and *S. chienesis* species*.*


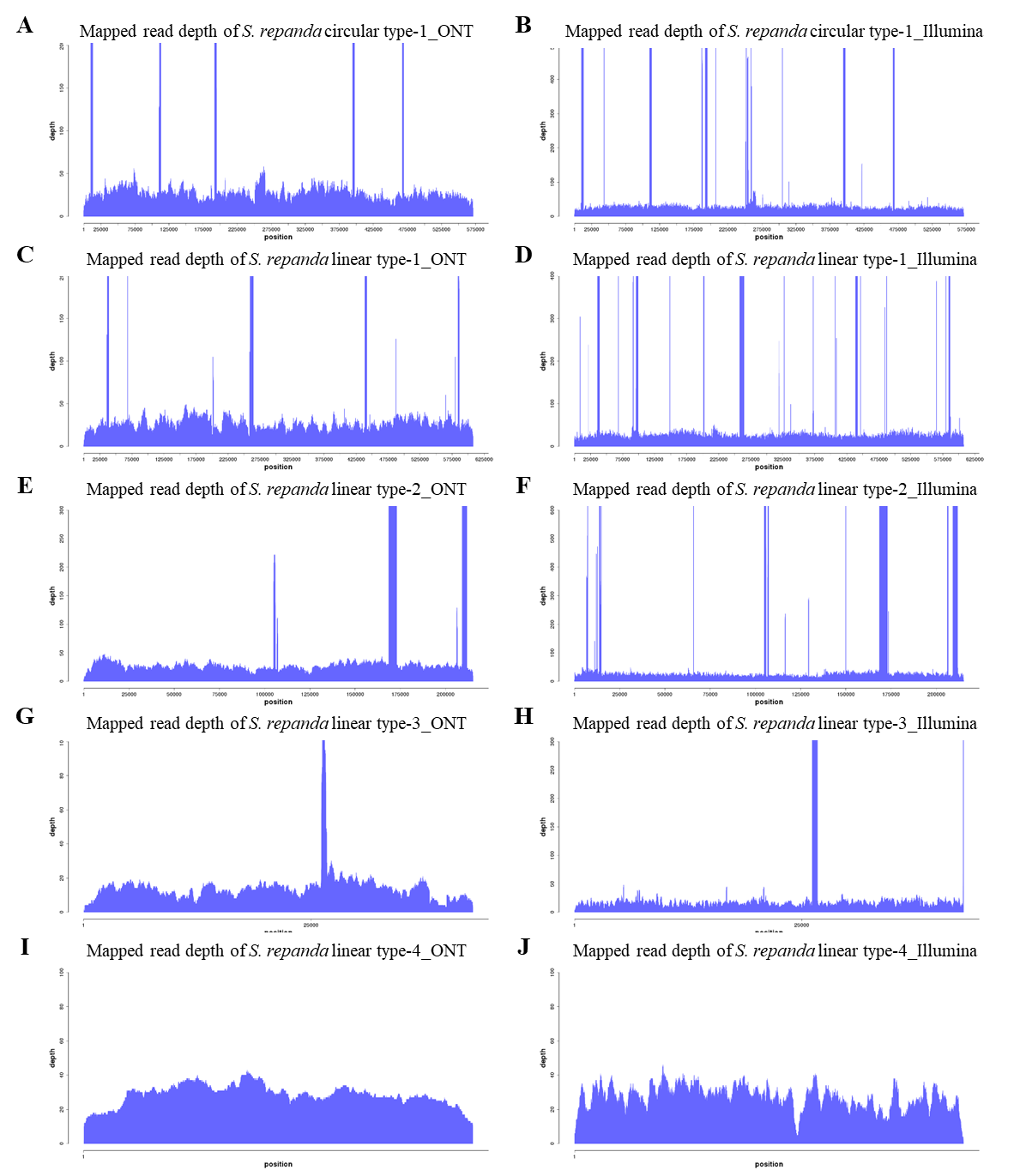


**Supplementary Figure 1.** Mitogenome depth-read histograms from ONT and Illumina for *S. repanda* mitogenomes. (**A-B**) *S. repanda* circular-1. (**C-D**) *S. repanda* linear-1. (**E-F**) *S. repanda* linear-2. (**G-H**) *S. repanda* linear-3. (**I-J**) *S. repanda* linear-4.

**
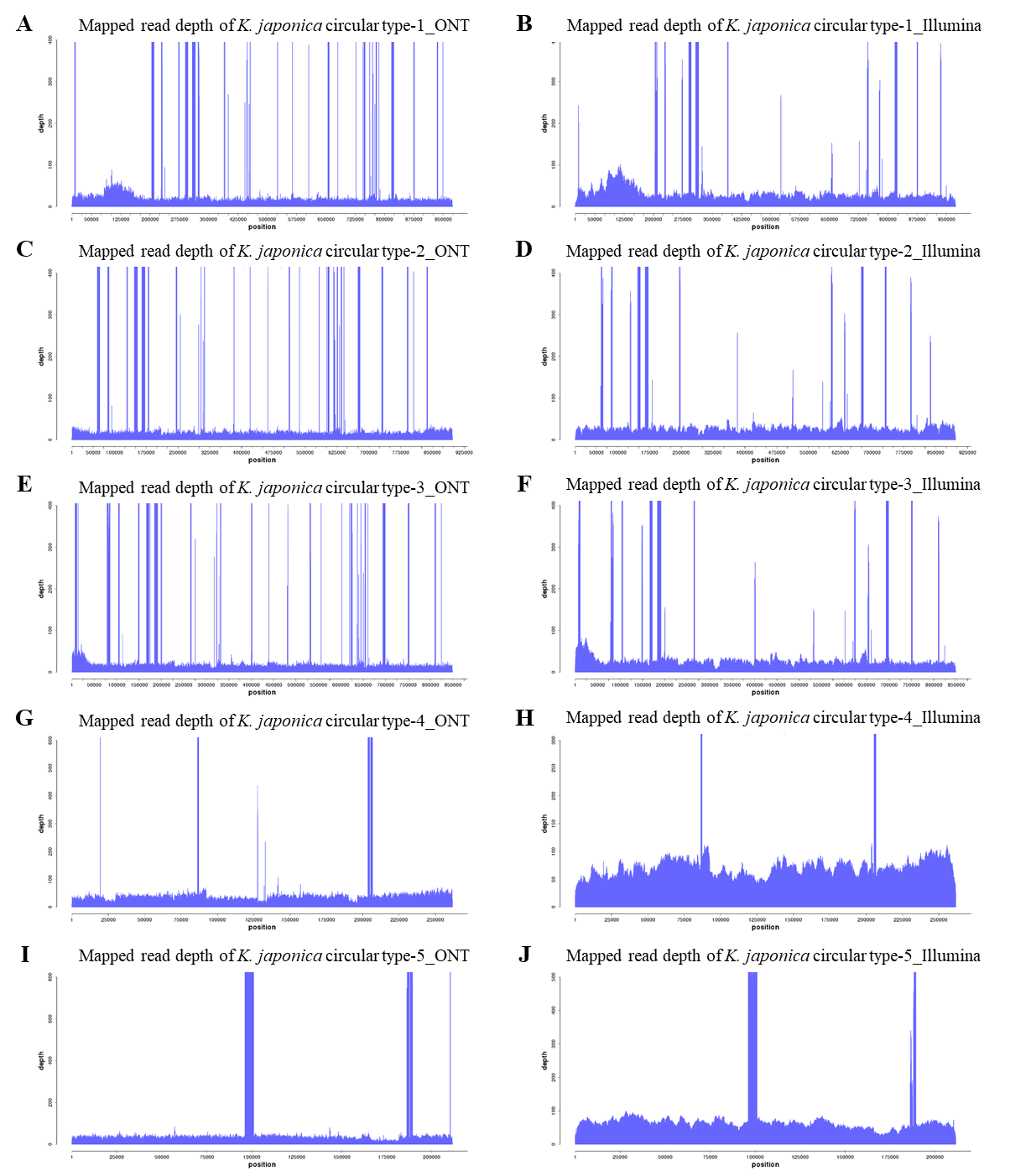
**

**Supplementary Figure 2.** Mitogenome depth-read histograms from ONT and Illumina for *K. japonica* mitogenomes. (**A-B**) *K. japonica* circular-1. (**C-D**) *K. japonica* circular-2. (**E-F**) *K. japonica* circular-3. (**G-H**) *K. japonica* circular-4. (**I-J**) *K. japonica* circular-5. (**K-L**) *K. japonica* linear-1. (**M-N**) *K. japonica* linear-2. (**O-P**) *K. japonica* linear-3.


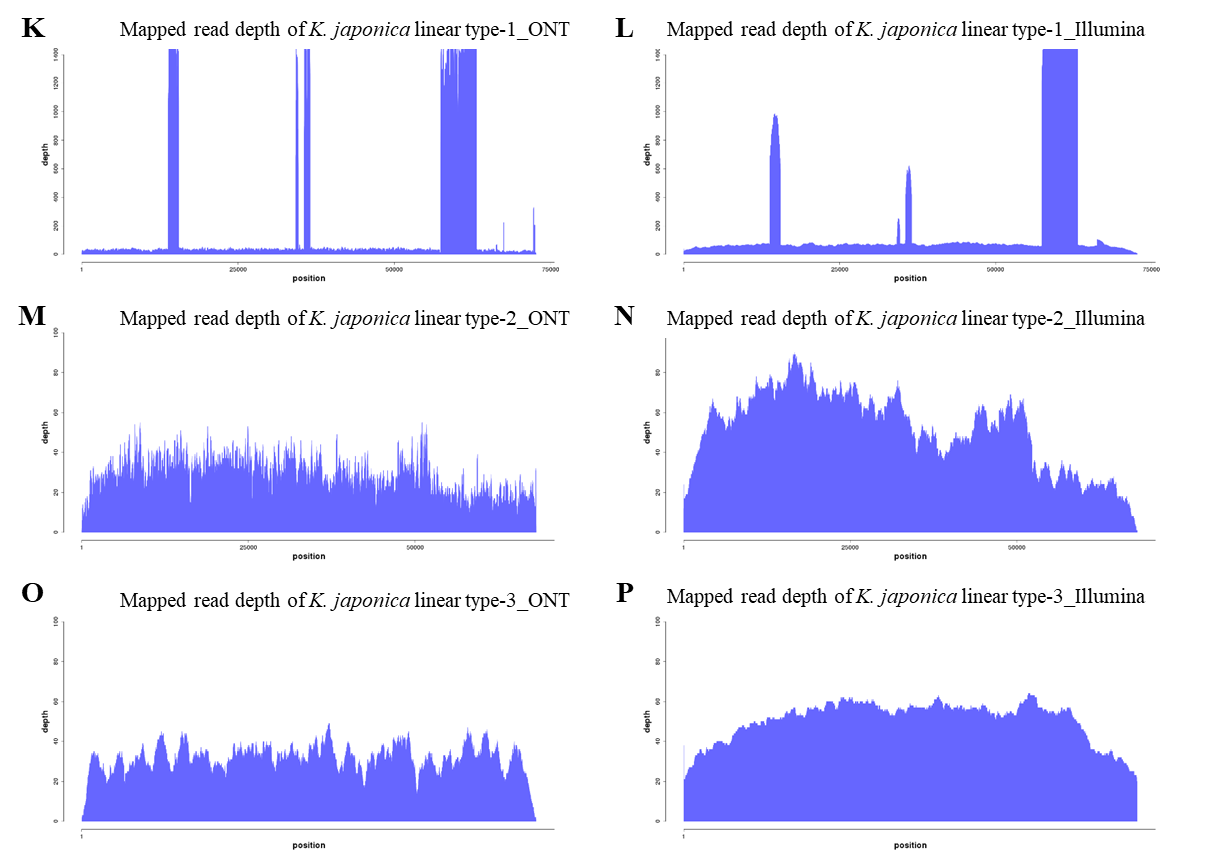


**Supplementary Figure 2.** Continued.


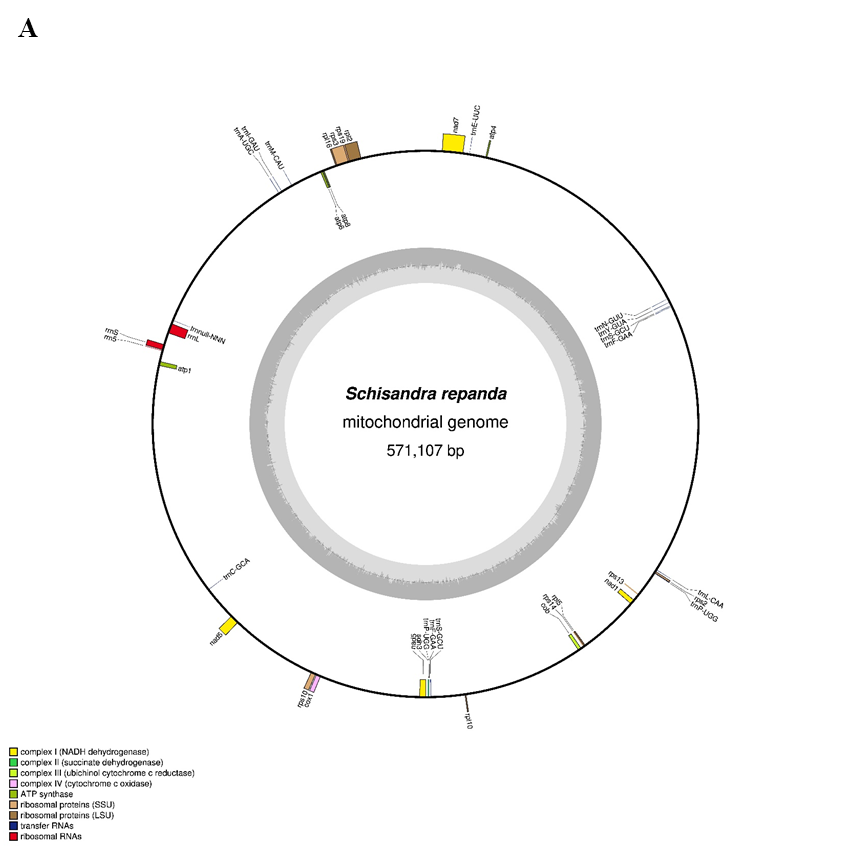


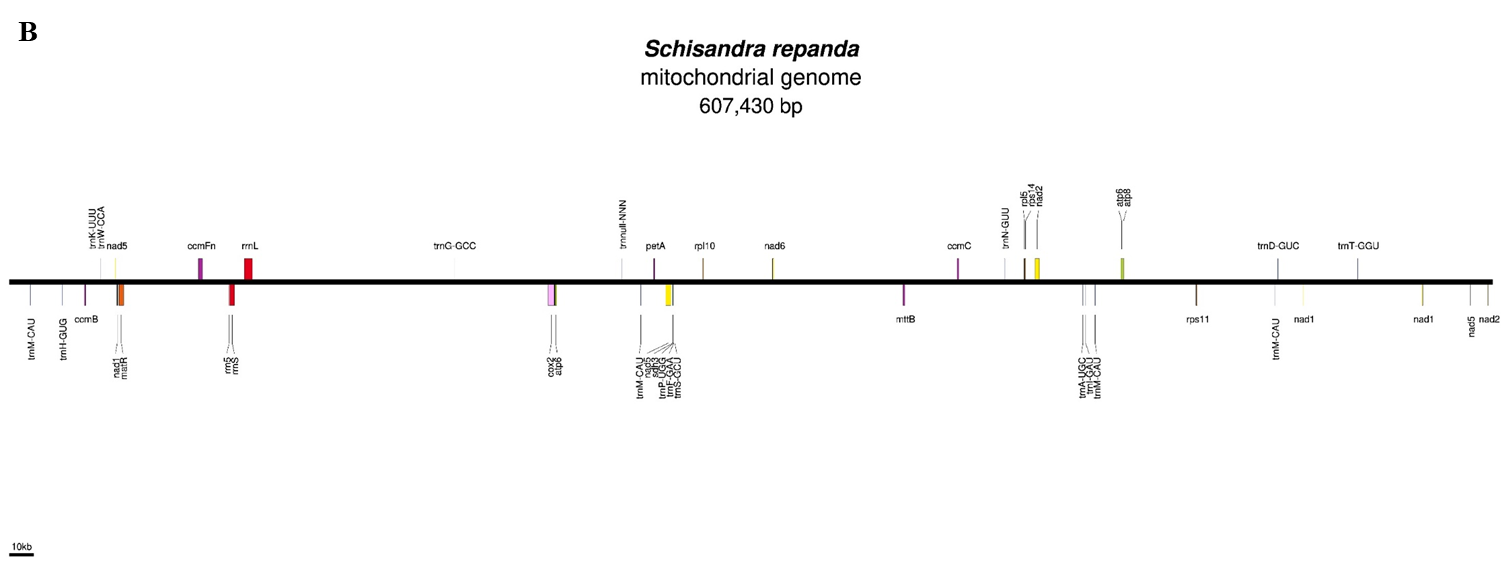


**Supplementary Figure 3.** Map of the five configs of *S. repanda* mitogenome. (**A**) Circular config (circular-1; 571,107 bp). (**B-E**) Linear configs (linear-1, 607,403 bp; linear-2, 215,128 bp; linear-3, 42,796 bp and linear-4, 10,898 bp).


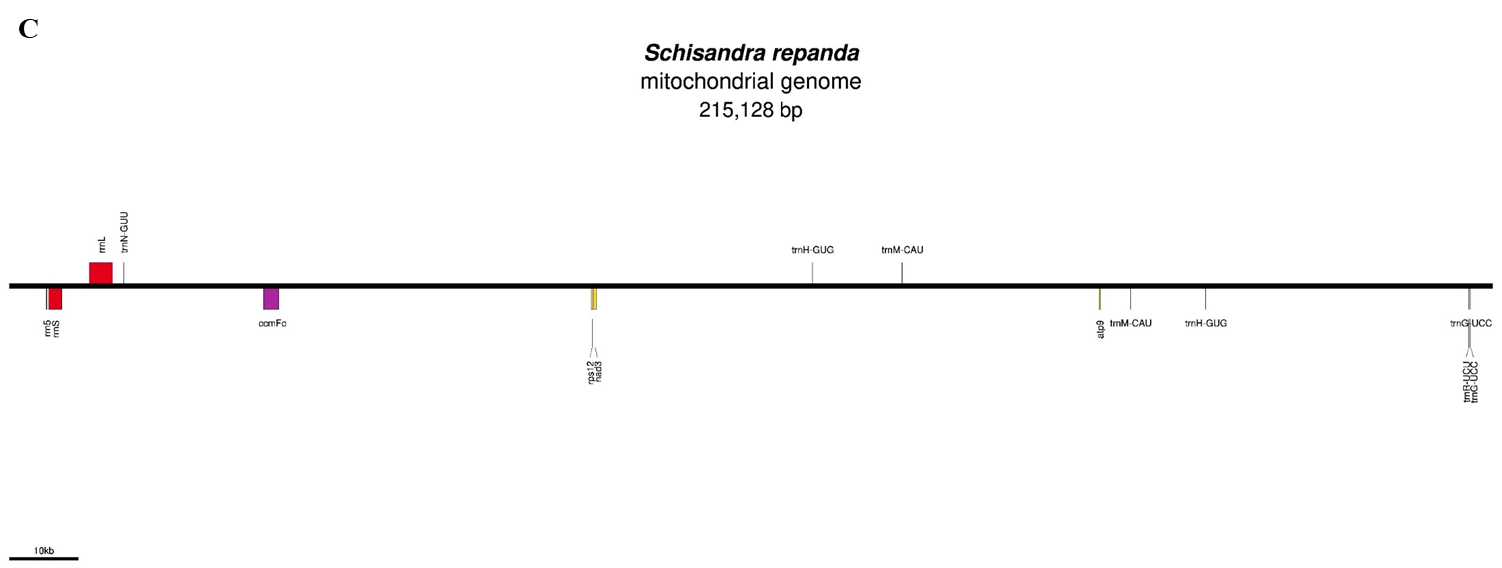

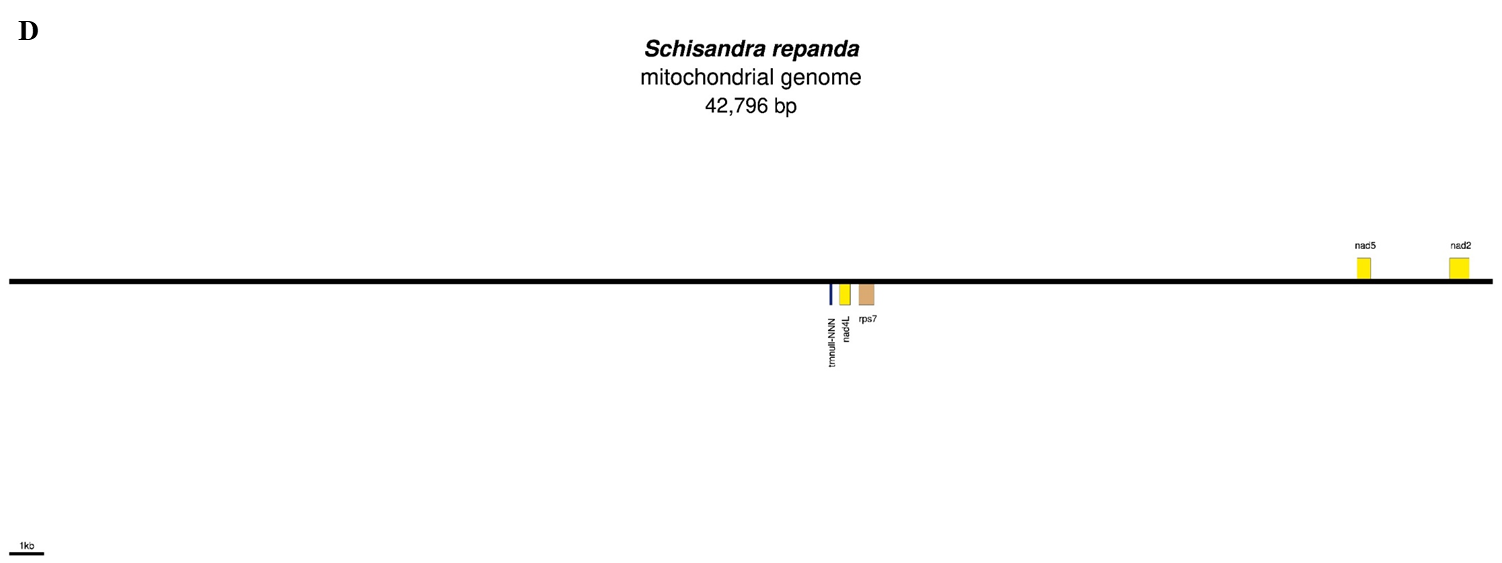

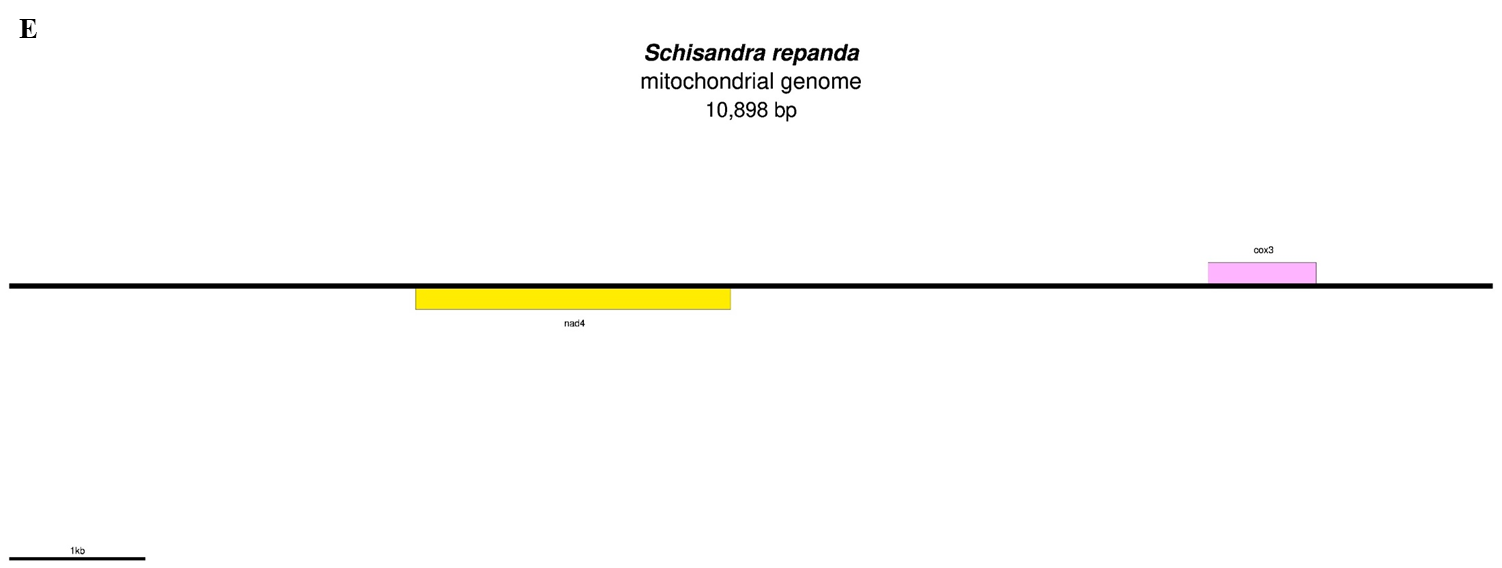


**Supplementary Figure 3.** Continued.

**
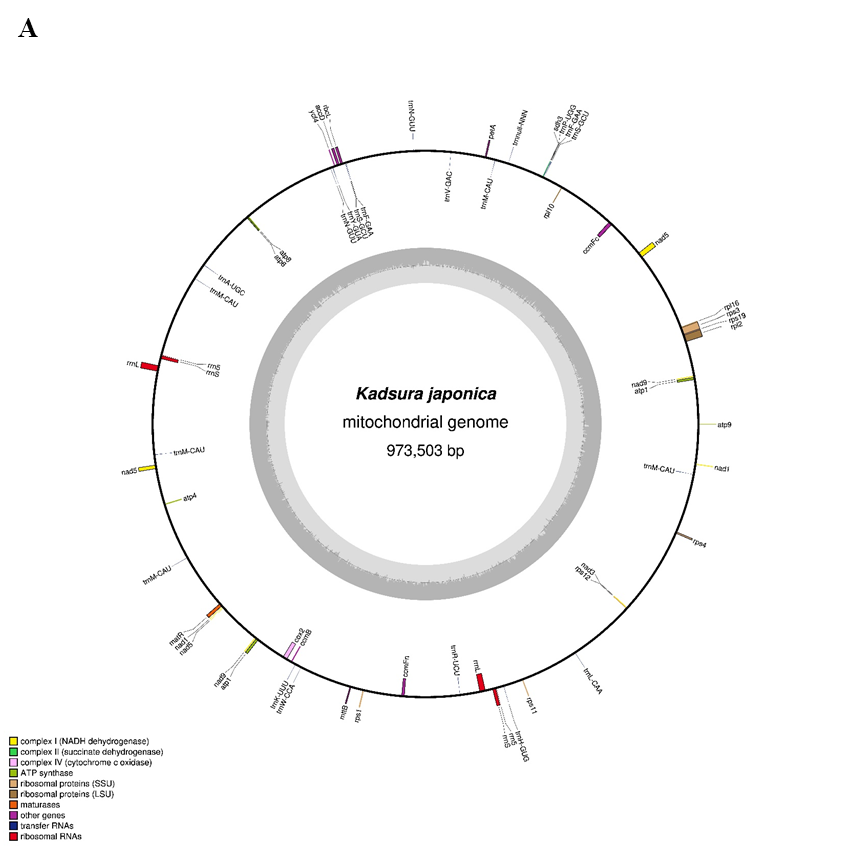
**

**Supplementary Figure 4.** Map of the eight configs of *K. japonica* mitogenome. (**A-E**) Circular configs (circular-1, 973,503 bp; circular-2, 897,204 bp; circular-3, 848,837 bp; circular-4, 261,590 bp; and circular-5, 211,474 bp). (**F-H**) Linear configs (linear-1, 72,712 bp; linear-2, 68,176 bp and linear-3, 8,010 bp).

**
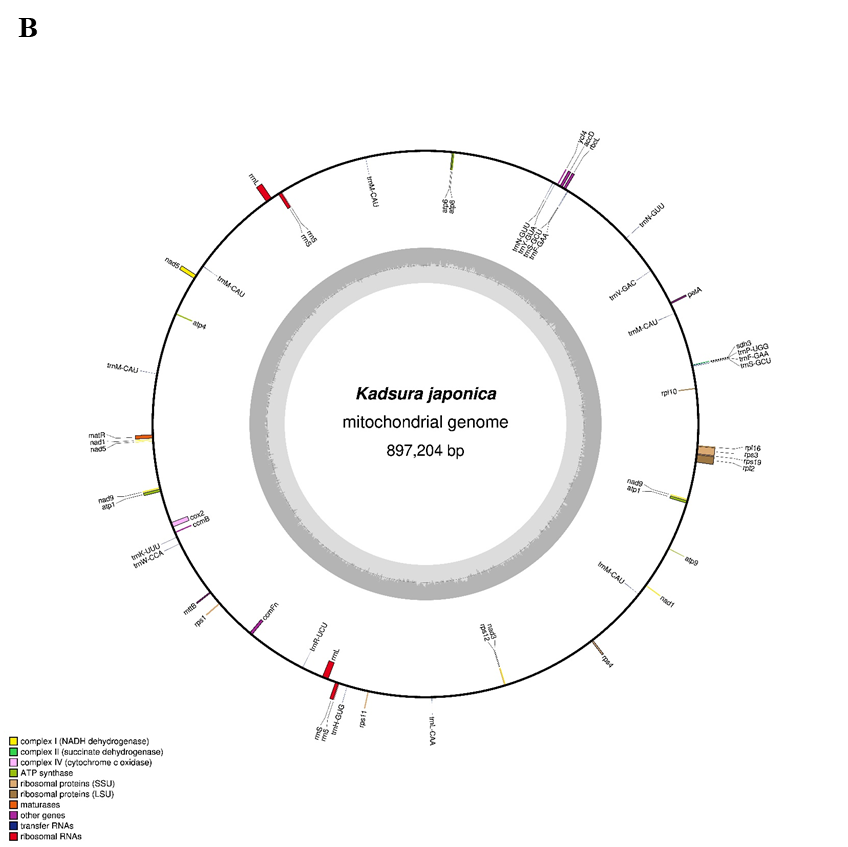

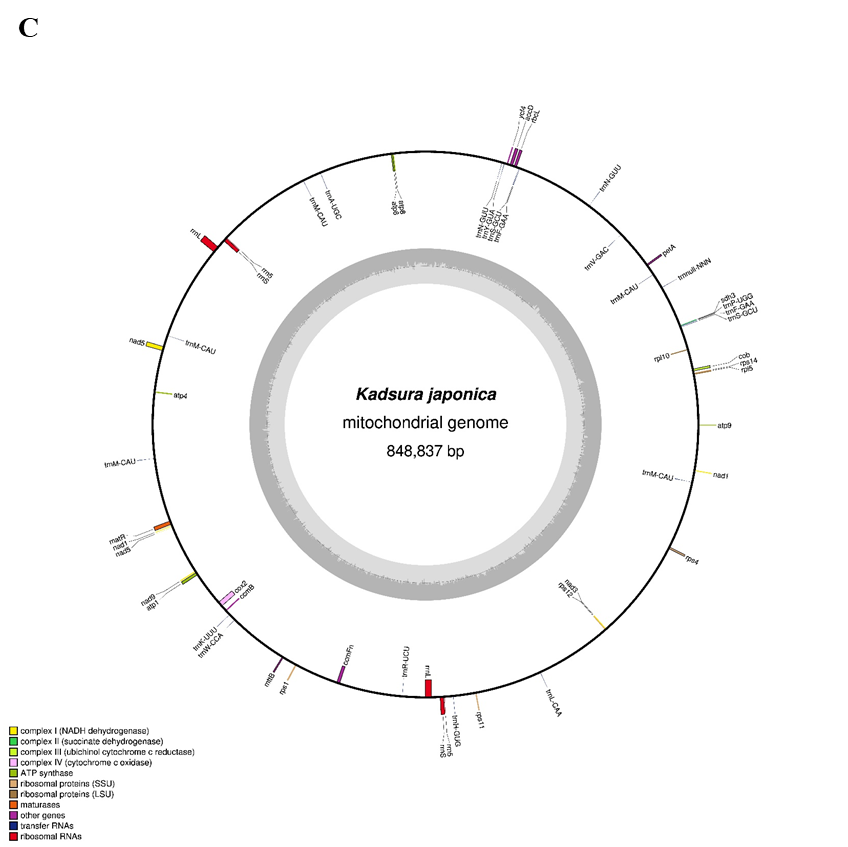
**

**Supplementary Figure 4.** Continued.


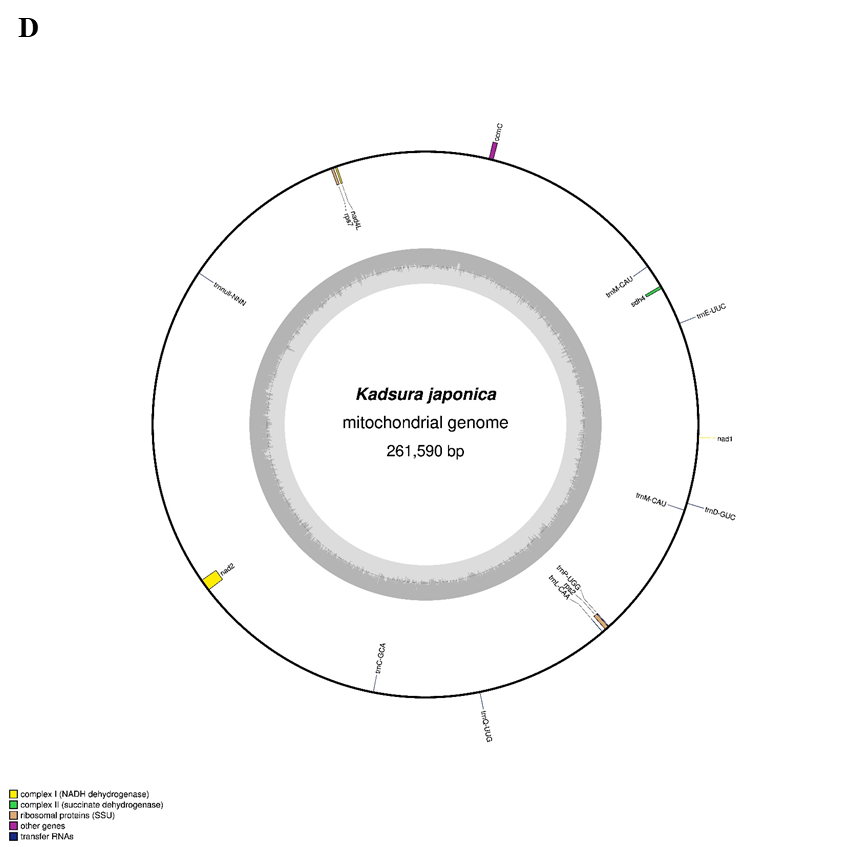

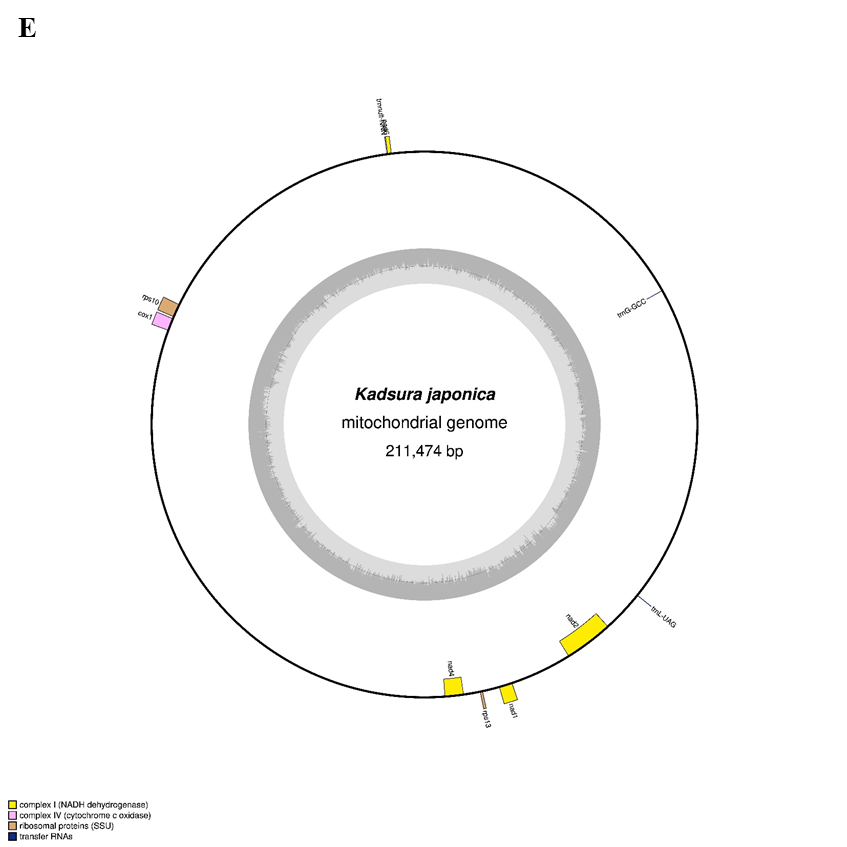


**Supplementary Figure 4.** Continued.


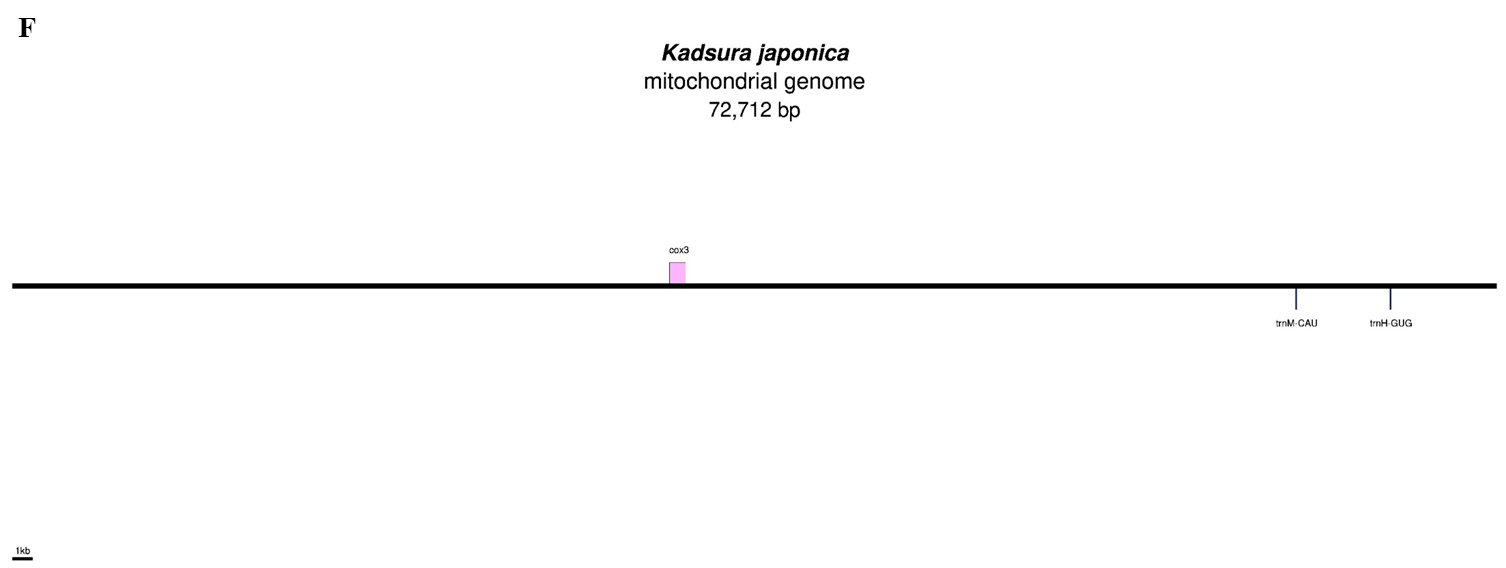


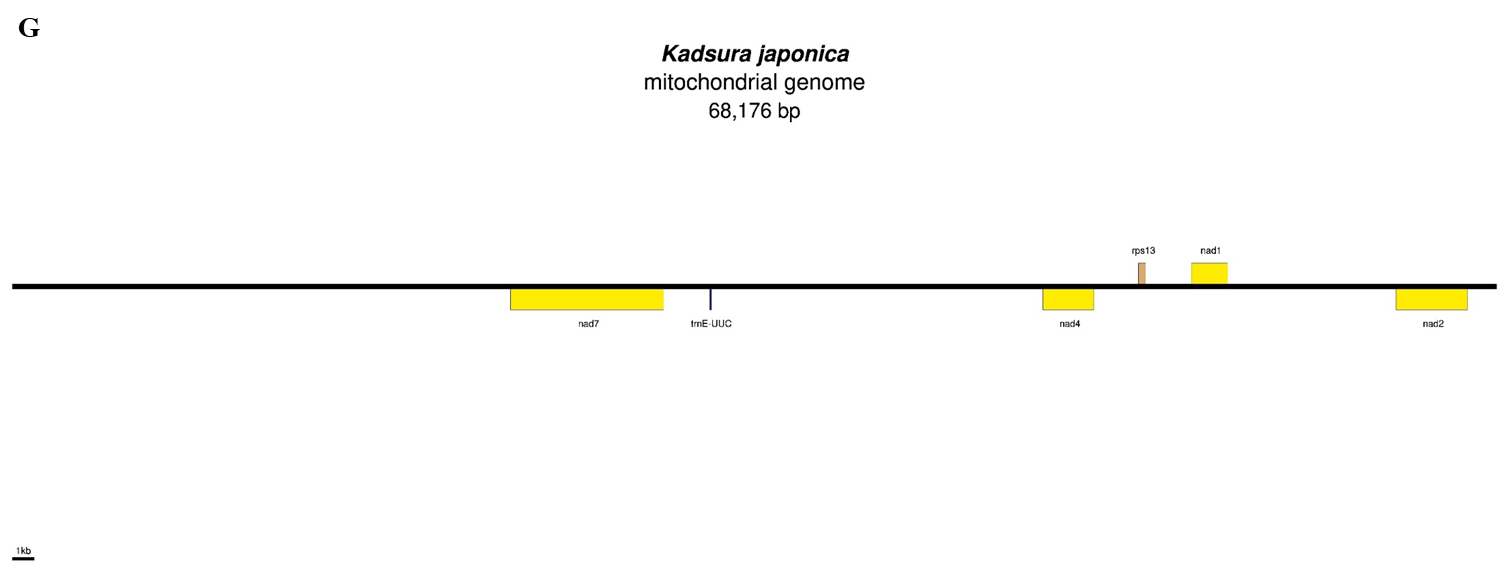

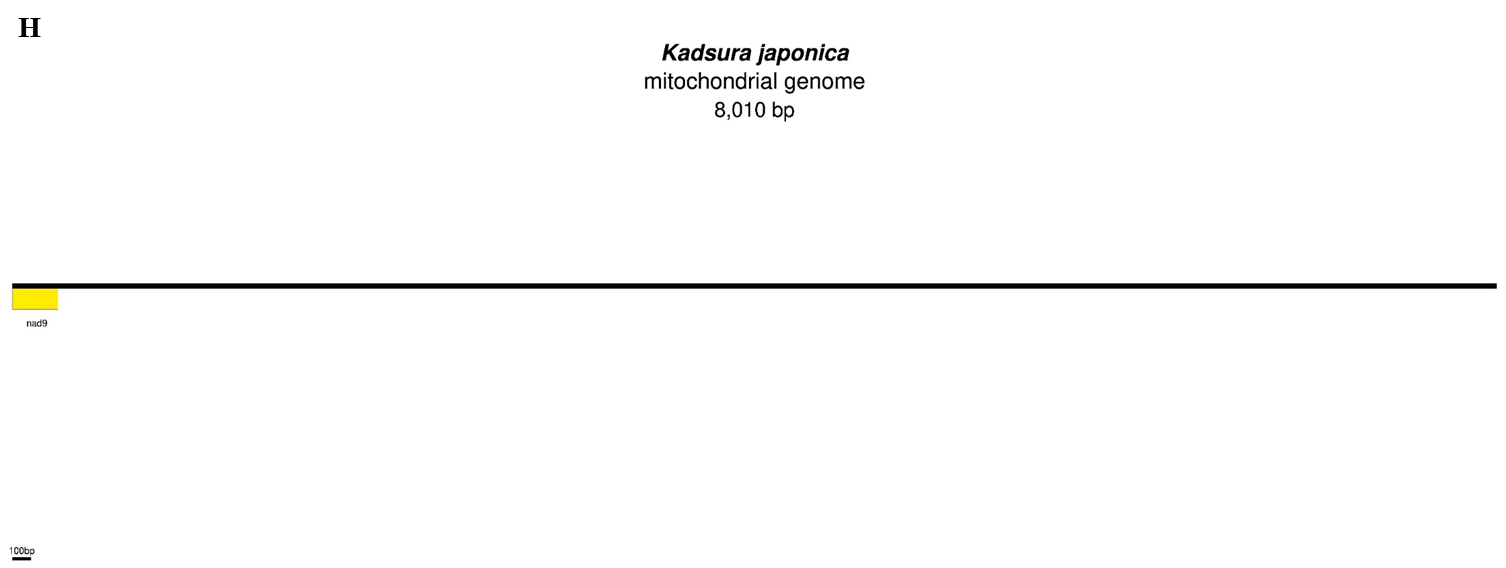


**Supplementary Figure 4.** Continued.

**
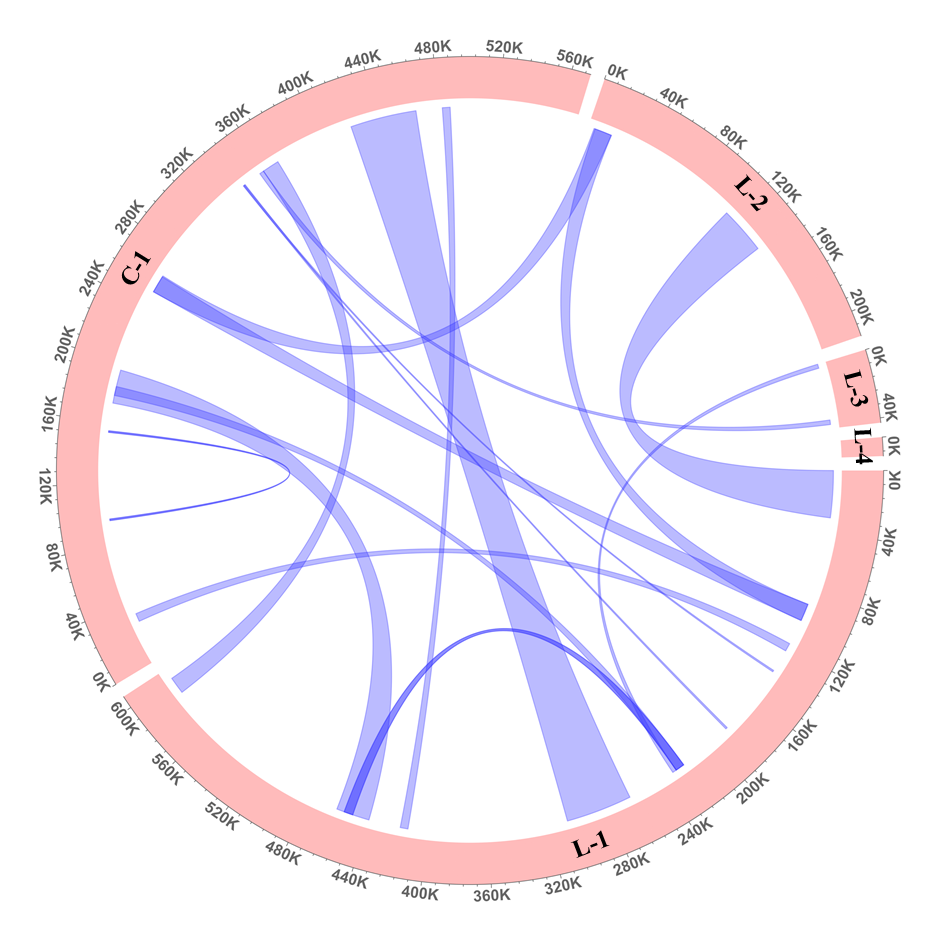
**

**Supplementary Figure 5.** Circos plot showing comparison of five *S. repanda* mitogenome contigs. The outer track (light pink) indicates *S. repanda* mitogenome contigs. Inner lines (light blue) represent regions with similarity among *S. repanda* mitogenome contigs identified by BLATN searches (task option megablast, cutoff e-value 1e-5, match length > 1 kb).

**
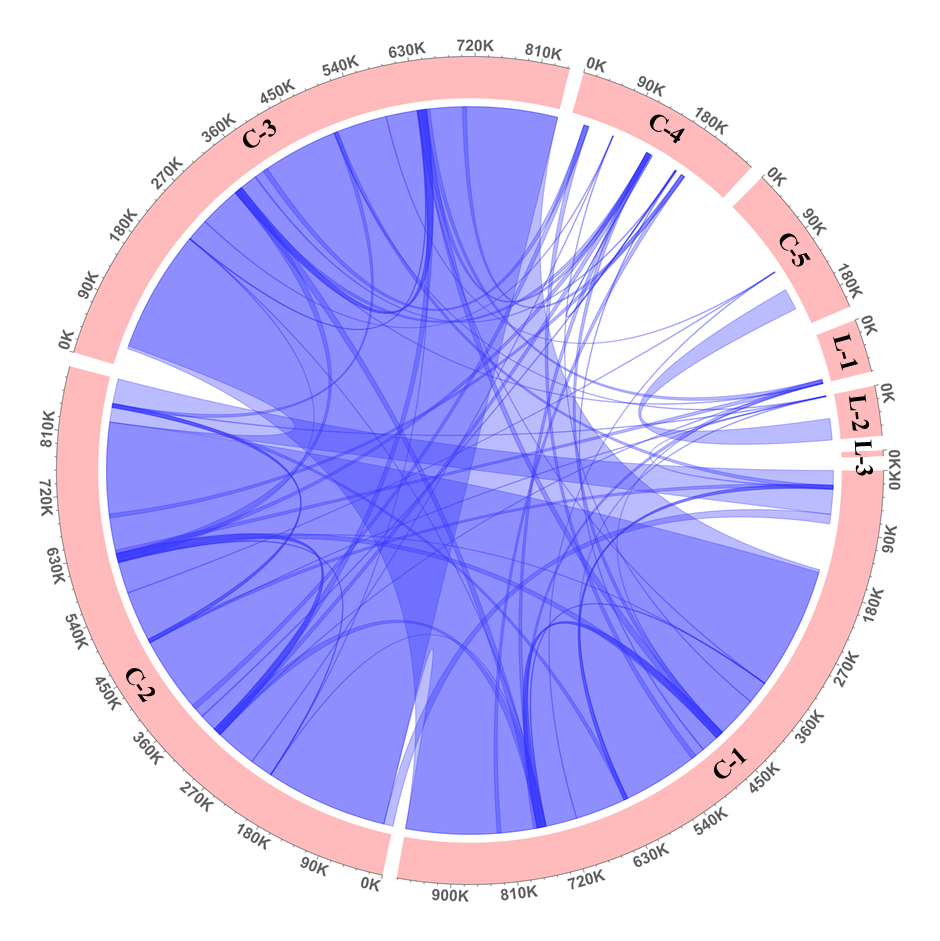
**

**Supplementary Figure 6.** Circos plot showing comparison of eight *K. japonica* mitogenome contigs. The outer track (light pink) indicates *K. japonica* mitogenome contigs. Inner lines (light blue) represent regions with similarity among *K. japonica* mitogenome contigs identified by BLATN searches (task option megablast, cutoff e-value 1e-5, match length > 1 kb).
